# Supplementary material for: Using telemedicine to improve early medical abortion at home (UTAH): a randomised controlled trial to compare telemedicine with in-person consultation for early medical abortion
Source: BMJ Open. 2023 Sep 13;13(9):e073630. doi: 10.1136/bmjopen-2023-073630 (PMC10870195; doi:10.1136/bmjopen-2023-073630)
Supplement: Supplementary data [file bmjopen-2023-073630supp001.pdf]

Supplemental Figure 1: Contents of medication pack

Abortion medications	Misoprostol 800micrograms sublingual/vaginal/buccal (per patient preference)
Analgesia	Dihydrocodeine 30mg<br>Women advised to purchase their own supply of paracetamol and ibuprofen.
Confirmation of abortion	Low Sensitivity Urine Pregnancy Test 1000iu at 14 days
Contraception	Pills, patches, rings and condoms could be supplied in pack.<br>Implants were provided at clinic visit and intrauterine methods provided at rapid access clinic, with bridging method offered.
Information	Detailed step-by-step information provided as written leaflet included in pack.

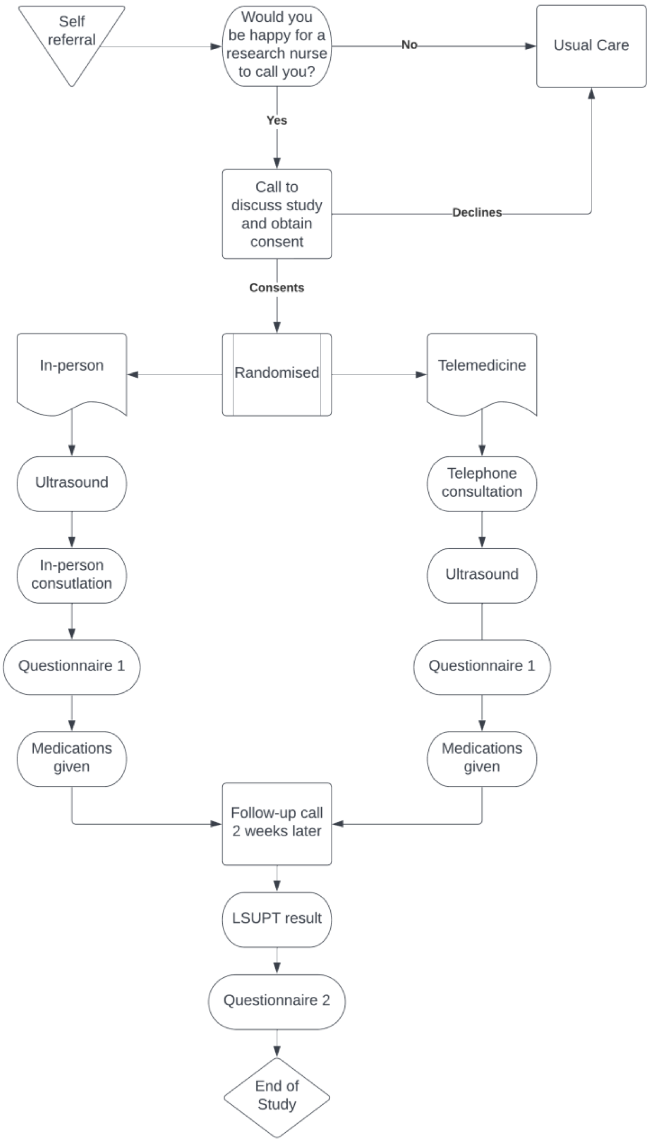

Supplemental Figure 2: Study flow chart
