# Supplementary material for: Using telemedicine to improve early medical abortion at home (UTAH): a randomised controlled trial to compare telemedicine with in-person consultation for early medical abortion
Source: BMJ Open. 2023 Sep 13;13(9):e073630. doi: 10.1136/bmjopen-2023-073630 (PMC10870195; doi:10.1136/bmjopen-2023-073630)
Supplement: Author's manuscript [file bmjopen-2023-073630.draft_revisions.pdf]

BMJ Open is committed to open peer review. As part of this commitment we make the peer review history of every article we publish publicly available.

When an article is published we post the peer reviewers' comments and the authors' responses online. We also post the versions of the paper that were used during peer review. These are the versions that the peer review comments apply to.

The versions of the paper that follow are the versions that were submitted during the peer review process. They are not the versions of record or the final published versions. They should not be cited or distributed as the published version of this manuscript.

BMJ Open is an open access journal and the full, final, typeset and author-corrected version of record of the manuscript is available on our site with no access controls, subscription charges or pay-per-view fees (http://bmjopen.bmj.com).

If you have any questions on BMJ Open's open peer review process please email info.bmjopen@bmj.com

BMJ Open

UTAH: Using Telemedicine to improve early medical Abortion at Home – a randomised controlled trial to compare telemedicine with in-person consultation for early medical abortion

Journal:	*BMJ Open*
Manuscript ID	bmjopen-2023-073630
Article Type:	Original research
Date Submitted by the Author:	13-Mar-2023

Complete List of Authors:	Reynolds-Wright, John; University of Edinburgh; NHS Lothian<br>Norrie, John; Edinburgh Clinical Trials Unit, University of Edinburgh No. 9, Bioquarter, Usher Institute<br>Cameron, Sharon; University of Edinburgh, Obstetrics and Gynaecology; NHS Lothian, Sexual and Reproductive Health

Keywords:	Telemedicine < BIOTECHNOLOGY & BIOINFORMATICS, GYNAECOLOGY, OBSTETRICS, SEXUAL MEDICINE, REPRODUCTIVE MEDICINE

SCHOLARONE™
Manuscripts

I, the Submitting Author has the right to grant and does grant on behalf of all authors of the Work (as defined in the below author licence), an exclusive licence and/or a non-exclusive licence for contributions from authors who are: i) UK Crown employees; ii) where BMJ has agreed a CC-BY licence shall apply, and/or iii) in accordance with the terms applicable for US Federal Government officers or employees acting as part of their official duties; on a worldwide, perpetual, irrevocable, royalty-free basis to BMJ Publishing Group Ltd ("BMJ") its licensees and where the relevant Journal is co-owned by BMJ to the co-owners of the Journal, to publish the Work in this journal and any other BMJ products and to exploit all rights, as set out in our licence.

The Submitting Author accepts and understands that any supply made under these terms is made by BMJ to the Submitting Author unless you are acting as an employee on behalf of your employer or a postgraduate student of an affiliated institution which is paying any applicable article publishing charge ("APC") for Open Access articles. Where the Submitting Author wishes to make the Work available on an Open Access basis (and intends to pay the relevant APC), the terms of reuse of such Open Access shall be governed by a Creative Commons licence – details of these licences and which Creative Commons licence will apply to this Work are set out in our licence referred to above.

Other than as permitted in any relevant BMJ Author's Self Archiving Policies, I confirm this Work has not been accepted for publication elsewhere, is not being considered for publication elsewhere and does not duplicate material already published. I confirm all authors consent to publication of this Work and authorise the granting of this licence.

For peer review only

Title

UTAH: Using Telemedicine to improve early medical Abortion at Home – a randomised controlled trial to compare telemedicine with in-person consultation for early medical abortion

Authors

John J Reynolds-Wright MBChB, PhD, MFSRH [1,2]
John Norrie MSc [3]
Sharon T Cameron MD FFSRH FRCOG [1,2]

Affiliations

[1]MRC Centre for Reproductive Health, University of Edinburgh, Edinburgh, UK
[2]Chalmers Centre, NHS Lothian, Edinburgh, UK
[3]Edinburgh Clinical Trials Unit, Usher Institute, University of Edinburgh, Edinburgh, UK

Correspondence:

Dr John J Reynolds-Wright
MRC Centre for Reproductive Health
The Queen's Medical Research Institute
47 Little France Crescent
Edinburgh BioQuarter
Edinburgh
EH16 4TJ
United Kingdom
Email: John.Reynolds-Wright@ed.ac.uk

For peer review only

Conflicts of Interest

JRW has received an educational grant from Gedeon Richter. STC and JN have none to declare.

Funding

This work was supported by the Edinburgh Family Planning Trust grant number EFPT/2019/UTAH. The project was conducted from the MRC Centre for Reproductive Health, supported by the Medical Research Council (grant MR/N022556/1).

Contributions

STC conceived the study. JRW, JN and STC designed the study protocol. JRW designed the study tools and led data collection. JRW, JN and STC reviewed and analysed the data. JRW wrote the initial manuscript. JRW, JN and STC all contributed to the final manuscript.

Data availability

Due to the sensitive nature of the data and small sample size, individual patient level data are not available publicly.

For peer review only

Abstract – total = 297/300 words

Objectives: To compare telephone consultations with in-person consultations for the provision of medical abortion (using Mifepristone 200mg and Misoprostol 800microgram). We hypothesised that telemedicine consultations would be non-inferior to in-person consultations with a non-inferiority limit of 3%.

Design: Randomised controlled trial with 1:1 allocation.

Setting: Community abortion service housed within an integrated sexual and reproductive health service.

Participants: The trial began 13 January 2020. Recruitment was suspended 31 March 2020 due to COVID-19 and formally closed 31 August 2021. A total of 125 participants were randomised, approximately 10% of the total planned, 63 telemedicine and 62 in-person consultation.

Primary Outcome: efficacy of medical abortion, defined as complete abortion without surgical intervention.

Secondary Outcomes: satisfaction with consultation type, preparedness , unscheduled contact with care, complication rate, time spent in clinical contact and uptake of long-acting contraception.

Results: Primary outcome was available for 115 participants (lost-to-follow-up telemedicine=2, in-person=8), secondary outcomes were available for 110 participants (n=5 and n= 10 in telemedicine and in-person groups did not complete questionnaires). There were no significant differences between groups in treatment efficacy (telemedicine 57/63 (90.5%), in-person 48/62 (77.4%) However non-inferiority was not demonstrated (+3.3% in favour of telemedicine, confidence interval -6.6% to +13.3%, lower than non-inferiority margin). There were no significant differences in most secondary outcomes, however, there was more unscheduled contact with care in the telemedicine group (12(19%) vs 3(5%), p=0.01). The overall time spent in clinical contact was statistically significantly lower in the telemedicine group (mean (SD) 94 (24) vs 111 (24) minutes, p=0.0005).

Conclusions: Telemedicine for medical abortion appeared to be effective, safe, and acceptable to women but with less time spent in the clinic. However, due to the small sample size the study was underpowered to confirm this. These findings warrant further investigation in larger scale studies.

Registration: https://clinicaltrials.gov/ct2/show/NCT04139382

For peer review only

Strengths and Limitations of this study

- This is the first RCT in a high-income setting to compare telemedicine with in-person consultation for the delivery of early medical abortion care.
- The study was designed in line with the CONSORT and MARE guidance. Most other studies of telemedicine in abortion care have been observational cohort studies and so this trial makes a useful addition to the literature.
- Due to COVID-19, the UTAH study had to close shortly after recruitment began as telemedicine became the standard of care and randomising women to in-person appointments was deemed unsafe for COVID-19 transmission. As a result, only 10% of the target sample was recruited and the study outcomes are underpowered.

For peer review only

Introduction

In-person consultations prior to abortion can be lengthy. Women can struggle with arranging childcare or time off work to attend appointments. There is evidence from observational studies that telemedicine consultations for those seeking early medical abortion (EMA) at under 10 weeks gestation may be a safe and acceptable alternative (1–5).
Additionally, the World Health Organization recommend that women can reliably self-manage much of EMA with support from a healthcare worker (6).

Prior to COVID-19 in Scotland, women who chose an EMA typically made a single visit to a clinic for a consultation, assessment of gestation, receipt of mifepristone (to be administered in clinic, as per UK legal requirements at the time) and a medication pack to take home. This contained misoprostol (to self-administer at home contraception where desired, and instructions on how to self-assess the success of the abortion using a self-performed low-sensitivity urinary pregnancy test (LSUPT, detection limit 1000 iu human chorionic gonadotrophin) at home two weeks later (7–9).

This clinic visit could last up to two hours; much of which may be time spent in the waiting room between seeing clinicians and having an ultrasound to confirm gestation. However, a significant proportion of consultation time is standardised history taking and information giving, and could be conducted via telemedicine by telephone, video call, or over the internet, rather than in-person.

Telemedicine consultations could add flexibility for women (e.g. consultation from home), and benefit services with reduced footfall in clinics and allow for more flexible staff working (including remote working). It is also possible that women may be better prepared for EMA: having a telemedicine consultation in advance of receipt of medications may mean they have had more time to process the information provided about EMA, as well as any information about post-abortion contraception options. Doing this prior to attending clinic for ultrasound (which was nearly universal prior to COVID-19) and treatment provision, may give further opportunity to clarify areas of uncertainty for abortion treatment or select a contraceptive method.

We therefore aimed to compare telemedicine with in-person consultations regarding effectiveness, safety, acceptability, and duration of consultation.

Methods

The full study protocol has been published online (10). In brief, we designed a randomised controlled trial to compare telemedicine consultations, specifically a telephone call, with in-person consultations for women seeking EMA.

Participants were pregnant individuals seeking medical abortion at home, aged 16 or over, able to communicate in English and fulfil the requirements of the 1967 Abortion Act. In addition to being eligible to participate in the trial, women needed to be clinically eligible to receive EMA, which for the study was defined as having a viable

For peer review only

intrauterine pregnancy determined by transabdominal or transvaginal ultrasound at or less than 9 weeks and 6 days' gestation.

The study was conducted at a single centre in Scotland (Chalmers Centre, Edinburgh) that provides abortion care to a predominately urban population with some women living in more remote and rural areas. Standard care was for women to self-refer to the service by telephone and receive a subsequent in-person clinic appointment (11). At this appointment, they had a routine pre-abortion ultrasound scan to determine location and gestation of the pregnancy, followed by a consultation with a doctor or nurse to obtain medical history, discuss the chosen method of abortion and provide written informed consent. For medical abortion at home, the first medication (mifepristone 200mg) was provided orally in the clinic as per UK legal requirements at that time. Women were then provided with a medication pack for home use that included misoprostol 800 micrograms (sublingual or vaginal as per patient preference), contraceptive supplies if desired, and LSUPT and written information were provided to take home (9,12) (see supplemental figure 1).

In the study, at the point of self-referral for abortion, the clinic administrative team asked women if they would be happy to be contacted by a research nurse. Those agreeing received a telephone call the same or next day, during which the clinical research nurses explained the study, obtained consent and randomised women using REDCap software, hosted by University of Edinburgh (13,14). The randomisation list was produced by an independent statistician. Due to the nature of the interventions, blinding was not possible.

The 'in-person' group then went on to receive standard care as described above. Following this in-person consultation they completed a short interviewer-administered questionnaire with the research nurse in clinic.
The 'telemedicine' group received their telephone consultation using the same proforma as for in-person consultations. This was conducted by the research nurses, who has experience working in the abortion clinic and providing counselling in in-person consultations. This was done either immediately after randomisation or at another time, as preferred by the woman. Following this consultation, the woman then attended the clinic for ultrasound scan, medication provision and completed the study questionnaire (as for in-person care group). Women in both groups received a follow up telephone call at two weeks after EMA from a research nurse to determine the LSUPT result and to administer the follow up questionnaire. These questionnaire data were combined with routinely collected clinical data on demographics, gestation as determined by pre abortion ultrasound reproductive history and deprivation score (Scottish Index of Multiple Deprivation, SIMD (15)). See supplemental figure 2 for overview of study flow.

The primary outcome of this study was to determine if EMA conducted following a telemedicine consultation is as effective (complete abortion rate without surgical intervention) as following in-person consultation. Effectiveness was chosen in accordance with the Medical Abortion Reporting of Efficacy (MARE) core outcome recommendations (16). This was determined in the first instance using self-reported LSUPT result 2 weeks after EMA, any woman with a positive LSUPT result at this

For peer review only

point was invited to attend for follow up ultrasound to confirm/exclude continuing pregnancy. Any woman with a positive LSUPT but whose post abortion ultrasound showed an empty uterus was classed as having a complete abortion. The clinical database was reviewed at 6 weeks post-EMA to check for any admission or surgical intervention to a hospital within the region.

The secondary outcomes were total time spent in clinical contact to receive EMA (calculated as time spent in the clinic plus for the telemedicine group this also included the duration of the telephone consultation), preparedness for EMA as assessed by pre-abortion questionnaire (published as supplemental file to the open access protocol (10)), level of satisfaction with consultation as assessed by follow up questionnaire at 2 weeks (published as supplemental file to the open access protocol (10)), rates of unscheduled contact with abortion service and any complications resulting from abortion treatment (by case note review at 6 weeks post EMA), proportion of women who were found to be ineligible for EMA when attending clinic for pre-abortion ultrasound.

Statistical methods

The recruitment target of 1222 was calculated for the primary outcome, success of EMA, using a binary outcome non-inferiority design assuming an EMA success rate of 97% in both telemedicine and in-person groups, with 90% power, one-sided 5% level of significance, 3% non-inferiority limit, 1:1 allocation and around 10% compensation for loss to follow-up(17). Primarily due to the COVID-19 pandemic, the study only recruited 125 participants, and the observed EMA rate in the standard of care group was lower at 90% rather than the assumed 97%. Indicatively, a study with 125 evaluable participants and assumed EMA 90% could only detect a non-inferiority margin of almost 16% at 90% power and 1-sided 5% level of significance.

Descriptive statistics were used to characterise participants and assess comparability of the two groups at baseline.

The primary outcome of complete abortion was compared between the randomised groups (telemedicine vs in-person) by calculating the risk difference and its exact 95% confidence interval (risk difference calculator, neoweb.org.uk).

Secondary outcomes were analysed using appropriate tests depending on distribution of the data: for continuous data, we have used two sample t-tests and Mann Whitney tests; for categorical data, we have used chi-squared and Fisher Exact tests. We have not imputed any missing data for the secondary outcomes. Results will be considered statistically significant if p value <0.05. There has been no adjustment for multiple comparisons.

Ethical approval

Ethical approval was granted by South East Scotland NHS Research Ethics Committee on 28 October 2019, reference: 19/SS/0111.

For peer review only - http://bmjopen.bmj.com/site/about/guidelines.xhtml

The trial was prospectively registered with clinicaltrials.gov on 25 October 2019 (unique identifier: NCT04139382 URL: https://clinicaltrials.gov/ct2/show/NCT04139382)

Early cessation

The study opened to recruitment on 13 January 2020 and was suspended on 31 March 2020 in response to COVID-19 pandemic, as telemedicine became standard care. The study was formally closed on 31 August 2021 following the result of the Scottish public consultation on telemedicine for abortion (18).

Results

During the study period 575 people had abortions. Of these 128 met inclusion criteria and were approached, of whom 125 women were recruited - 63 (50.4%) in the telemedicine and 62 (49.6%) in the in-person group. Treatment outcome data were available 115 women (92%). 15 women (12%) did not complete follow up questionnaires (Telemedicine = 5, In-person = 10).

Demographics are summarised in table 1, there were no substantial differences between groups except for SIMD– the in-person group had a higher proportion of women from more deprived backgrounds.

Table 1: Demographics

Table 1	Telemedicine	In-person
BMI kg/ m2 /mean	26.4 (n = 56)	26.6 (n = 52)
Parity – total mean	1.2 (n =63)	1.6 (n=62)
Parity – live births (mean)	0.6 (n = 63)	0.98 (n = 62)
Age (mean)	28.2 (n= 63)	27.5 (n = 62)
Smoker (n/tot, %)	14/63, 22.2%	8/62, 12.9%
**Gestational age weeks + days (Median)**	6+6	6+5
**Gestational age weeks + days (Range)**	Less than 5 - 9+6	Less than 5 - 9+2
**Previous live birth (%)**	23 (36.5%)	29 (46.8%)
**Previous abortion (%)**	19 (30.2%)	15 (24.2%)
SIMD		
Unknown	1	1
1 (most deprived)	5	18
2	11	13
3	11	11

For peer review only

4	16	5
5 (least deprived)	19	14

Primary outcome

The complete abortion rate was high and not statistically significantly different between the two groups with a greater degree of loss to follow up in the in-person group (Table 2). The risk difference of failed abortion (continuing pregnancy) was 3.2% (CI -7.6 – 1.2). Since only around 10% of the target sample of 1220 was recruited, the study was generally substantially under powered.

Table 2: Treatment outcome

Table 2	Telemedicine	In-person
**Complete abortion (negative LSUPT or empty uterus on post-treatment scan)**	Of all enrolled: 57/63 (90.5%) Excluding loss to follow up: 57/61 (93.4%)	Of all enrolled: 48/62 (77.4%) Excluding loss to follow up: 48/54 (88.9%)
**Continuing Pregnancy after treatment**	0	2/62 (3.2%)
**Miscarriage prior to treatment**	1 (1.6%)	3 (4.8%)
**Not pregnant at clinic attendance**	2 (3.2%)	1 (1.6%)
**Ectopic on ultrasound**	1 (1.6%)	0
**Lost to Follow Up**	2/63 (3.2%)	8/62 (12.9%)

Secondary outcomes

There were no complications resulting from EMA in either the in-person or telemedicine group. No patients in either group required hospitalisation, blood transfusion, emergency department visits and there were no deaths. No patients were found to be ineligible for EMA in the telemedicine group when they attended for ultrasound (i.e. gestation was not higher than expected). A higher proportion of the telemedicine group reported a positive LSUPT and had a planned further attendance for repeat ultrasound to confirm complete abortion (telemedicine 12/63, 19% vs in-person 3/62, 4.8%), however this outcome was underpowered.

Following the consultation, similar high proportions of women rated themselves as very prepared and very satisfied with the mode of consultation they received for their abortion care. See table 3.

Table 3: Preparedness for treatment and Satisfaction with treatment

Table 3	Telemedicine N(%)	In-person

Preparedness		
Very Prepared	51 (81.0%)	44 (71.0%)
Somewhat Prepared	6 (9.5%)	8 (12.9%)
Neutral	1 (1.2%)	0
Somewhat Unprepared	0	0
Very Unprepared	0	0
Missing	5 (7.9%)	10 (15.9%)
Satisfaction		
Very Satisfied	56 (88.9%)	51 (82.3%)
Somewhat Satisfied	2 (3.2%)	1 (1.6%)
Neutral	0	0
Somewhat Unsatisfied	0	0
Very Unsatisfied	0	0
Missing	5 (7.9%)	10 (15.9%)

There was no statistically significant difference in the types of post-abortion contraception selected between groups when comparing long-acting reversible methods with all other methods; nor with short acting methods, condoms and no method; nor more effective methods (hormonal and intrauterine) with less effective methods. See table 4.

Table 4: Post-abortion contraception

Table 4	Telemedicine	In-person
Copper IUD	2 (3.2%)	1 (1.6%)
Hormonal IUD	3 (4.8%)	2 (3.2%)
Subdermal Implant	2 (3.2%)	6 (9.7%)
Injectable	6 (9.5%)	0
Combined pill	11 (17.5%)	11 (17.7%)
Combined patch	0	1 (1.6%)
Progestogen only Pill	9 (14.3%)	19 (30.6%)
Condoms	14 (22.2%)	8 (12.9%)
No method	16 (25.4%)	14 (22.6%)

The mean total time spent in clinical contact with the service (telemedicine consultation and clinic visit combined for the telemedicine group; clinic visit time for the in-person group) was significantly lower ($p<0.001$) in the telemedicine group compared to the in-person group. See table 5

Table 5: Time spent in clinical contact

For peer review only

Total Minutes Contact (minutes)	*Telemedicine (teleconsult duration only) n=55*	*Telemedicine (in clinic duration only) n = 55*	Telemedicine (teleconsult AND in clinic combined) n=55	In-person (time in clinic) N=50
Mean	*24*	*69*	**94**	111
St. Dev.	*6*	*23*	**24**	24
Median	*25*	*68*	**95**	108
Range	*10-40*	*25-125*	**55-151***	60-165

*Range of phone and in clinic combined is different from ranges in first two columns as those with shortest/longest call did not have shortest/longest clinic time

Discussion

This is the first RCT in a high income setting to compare telemedicine with in-person appointments for the delivery of abortion care. The study was designed in line with the CONSORT and MARE guidance(16). The majority of all other studies of telemedicine in abortion care have been observational cohort studies and so this trial makes a useful addition to the literature.

Due to COVID-19, the UTAH study had to close shortly after recruitment began as telemedicine became the standard of care and randomising women to in-person appointments was deemed unsafe for COVID-19 transmission. As a result the study outcomes are underpowered. However, the findings that effectiveness of treatment, satisfaction and preparedness are all similar between groups reinforces data from observational studies that telemedicine provision of abortion care is safe, effective, and acceptable to women.

The only adequately powered outcome was that time spent in pre-abortion clinical contact was significantly lower in the telemedicine group than in the in-person group. In a service utilising the model of telephone consultation followed by an ultrasound scan for gestational age and medication provision, the total clinical contact time may be even lower in practice still as in this study there were sometimes delays if telephone group patients got ‘stuck in the queue’ behind standard care patients in the clinic waiting for their ultrasound scan or medications. Models of care where an ultrasound is not required at all will likely reduce overall time in clinical care even further.

The rate of contact following abortion with a positive LSUPT was higher in the telemedicine group compared to the in-person group, however this outcome was underpowered and so may not have been borne out if the study had completed recruitment. If there was a true difference, this may possibly have resulted from greater familiarity and confidence in contacting the clinic via telephone as this was the main method these participants had used prior to abortion. While all patients were advised to make contact with the clinic if they had a positive or invalid result,

For peer review only - http://bmjopen.bmj.com/site/about/guidelines.xhtml

the telemedicine group may have been more confident in doing so, having received their prior care in this way.

The complete abortion rate in the in-person group (77%) is not statistically different from that of the telemedicine group but appears lower than reported elsewhere in the literature. This is likely to be due to the small numbers included in this study, meaning that this outcome is underpowered. Additionally, the participants that did not respond to follow-up contacts in this study arm did not have continuing pregnancies reported on their electronic patient record and so it is likely that they too had a complete abortion. The electronic patient record covers all the hospitals in the region and due to the geography of the area, patients uncommonly travel to neighbouring health boards.

The SIMD composition of the two study groups were different, with a greater proportion of the in-person group living in more deprived areas. This is likely to be due to chance and the small, under-recruited sample for the study.

In addition to this study, there is now a single RCT (19) in South Africa conducted since the start of COVID-19. In that study, those in the telemedicine arm had abdominal palpation from a trained nurse to assess fundal height and screen for pregnancies greater than 12 weeks' gestation. Last Menstrual Period was used to include patients with pregnancies less than 9 weeks' gestation. That study demonstrated non-inferiority of telemedicine to standard care with regard to complete abortion rates and a preference among women receiving abortion for telemedicine. The findings reflect the body of observational literature regarding telemedicine abortion care, including other observational cohort studies in the UK conducted during COVID-19 (20–22), and the findings of the UTAH study.

The legislation allowing medical abortion at home across Great Britain is now permanent (18,23), however many services in Great Britain and worldwide continue to provide abortion through in-person appointments. Moving assessment consultations to be done by telemedicine may result in time savings even if inviting the majority of patients for subsequent ultrasound.

We theorised that that this model of care may give women more time to consider contraceptive options and make a decision to select a more effective method than when confronted with this choice in a face-to-face clinic. However, in this trial as well as in subsequent observational cohort studies we have conducted at the same centre during COVID-19(24,25), this has not appeared to be the case. Further study is needed to better link women to effective post-abortion contraception, but this study indicates that women are not disadvantaged by receiving their abortion care via telemedicine.

Conclusion

For peer review only

Telemedicine consultations for early medical abortion appear to be effective and acceptable to women and result in less overall clinical contact time with abortion services.

References

1. Hyland P, Raymond EG, Chong E. A direct-to-patient telemedicine abortion service in Australia: Retrospective analysis of the first 18 months. Australian and New Zealand Journal of Obstetrics and Gynaecology [Internet]. 2018 Jun 1 [cited 2020 Nov 19];58(3):335–40. Available from: https://doi.org/10.1111/ajo.12800
2. Grossman D, Grindlay K. Safety of Medical Abortion Provided Through Telemedicine Compared With In Person. Obstetrics & Gynecology [Internet]. 2017;130(4). Available from: https://journals.lww.com/greenjournal/Fulltext/2017/10000/Safety_of_Medical_Abortion_Provided_Through.16.aspx
3. Grossman D, Grindlay K, Buchacker T, Lane K, Blanchard K. Effectiveness and Acceptability of Medical Abortion Provided Through Telemedicine. Obstetrics & Gynecology [Internet]. 2011;118(2 Part 1). Available from: https://journals.lww.com/greenjournal/Fulltext/2011/08000/Effectiveness_and_Acceptability_of_Medical.14.aspx
4. Wiebe ER. Use of telemedicine for providing medical abortion. Int J Gynaecol Obstet. 2014 Feb;124(2):177–8.
5. Kohn JE, Snow JL, Grossman D, Thompson TA, Seymour JW, Simons HR. Introduction of telemedicine for medication abortion: Changes in service delivery patterns in two U.S. states. Contraception [Internet]. 2021 Mar 1;103(3):151–6. Available from: https://www.sciencedirect.com/science/article/pii/S0010782420304583
6. World Health Organization. Expanding health worker roles for safe abortion in the first trimester of pregnancy [Internet]. Geneva; 2016 May. Report No.: WHO/RHR/16.02. Available from: https://www.who.int/reproductivehealth/publications/unsafe_abortion/health-worker-roles-safe-abortion-1st-trimester-summary/en/
7. Chief Medical Officer for Scotland. Abortion - Improvement to existing services - Approval for misoprostol to be taken at home [Internet]. SGHD/CMO(2017)14 Oct 26, 2017. Available from: https://www.sehd.scot.nhs.uk/cmo/CMO(2017)14.pdf
8. Cameron ST, Glasier A, Johnstone A, Dewart H, Campbell A. Can women determine the success of early medical termination of pregnancy themselves? Contraception [Internet]. 2015 Jan 1;91(1):6–11. Available from: http://www.sciencedirect.com/science/article/pii/S0010782414006908

For peer review only

9. Millar SL, Cameron ST. Comparison of two low-sensitivity urine pregnancy tests for confirming the success of early medical abortion. BMJ Sex Reprod Health [Internet]. 2018 Jan 1;44(1):54. Available from: http://jfprhc.bmj.com/content/44/1/54.abstract

10. Reynolds-Wright JJ, Norrie J, Cameron ST. UTAH: Using Telemedicine to improve early medical Abortion at Home: a protocol for a randomised controlled trial comparing face-to-face with telephone consultations for women seeking early medical abortion. BMJ Open [Internet]. 2021 Jun 1;11(6):e046628. Available from: http://bmjopen.bmj.com/content/11/6/e046628.abstract

11. Low ST, Chen ZE, Cameron S. Women's experiences of self-referral to an abortion service: qualitative study. BMJ Sex Reprod Health [Internet]. 2020 Apr 7;bmjsrh-2019-200568. Available from: http://jfprhc.bmj.com/content/early/2020/04/08/bmjsrh-2019-200568.abstract

12. Finch RE, McGeechan K, Johnstone A, Cameron S. Impact of self-administration of misoprostol for early medical abortion: a prospective observational cohort study. BMJ Sex Reprod Health [Internet]. 2019 Oct 1;45(4):296. Available from: http://jfprhc.bmj.com/content/45/4/296.abstract

13. Harris PA, Taylor R, Minor BL, Elliott V, Fernandez M, O'Neal L, et al. The REDCap consortium: Building an international community of software platform partners. Journal of Biomedical Informatics [Internet]. 2019 Jul 1;95:103208. Available from: https://www.sciencedirect.com/science/article/pii/S1532046419301261

14. Harris PA, Taylor R, Thielke R, Payne J, Gonzalez N, Conde JG. Research electronic data capture (REDCap)—A metadata-driven methodology and workflow process for providing translational research informatics support. Journal of Biomedical Informatics [Internet]. 2009 Apr 1;42(2):377–81. Available from: https://www.sciencedirect.com/science/article/pii/S1532046408001226

15. Scottish Government. Scottish Index of Multiple Deprivation (SIMD) [Internet]. 2020. Available from: www.simd.scot

16. Creinin MD, Chen MJ. Medical abortion reporting of efficacy: the MARE guidelines. Contraception. 2016;

17. Sealed Enveloped Ltd. Power calculator for binary outcome non-inferiority trial [Internet]. 2012. Available from: https://www.sealedenvelope.com/power/binary-noninferior/

18. Scottish Government. Early medical abortion at home - future arrangements: consutlation analysis [Internet]. 2021 Jun. Available from: https://www.gov.scot/publications/future-arrangements-early-medical-abortion-home-consultation-analysis/pages/1/

19. Endler M, Petro G, Gemzell Danielsson K, Grossman D, Gomperts R, Weinryb M, et al. A telemedicine model for abortion in South Africa: a randomised, controlled, non-inferiority trial. The Lancet [Internet]. 2022 Aug

For peer review only

27;400(10353):670–9. Available from: https://www.sciencedirect.com/science/article/pii/S014067362201474X

20. Aiken A, Lohr P, Lord J, Ghosh N, Starling J. Effectiveness, safety and acceptability of no-test medical abortion (termination of pregnancy) provided via telemedicine: a national cohort study. BJOG: An International Journal of Obstetrics & Gynaecology [Internet]. 2021 Feb 18 [cited 2021 Jun 25];n/a(n/a). Available from: https://doi.org/10.1111/1471-0528.16668

21. Reynolds-Wright JJ, Johnstone A, McCabe K, Evans E, Cameron S. Telemedicine medical abortion at home under 12 weeks' gestation: a prospective observational cohort study during the COVID-19 pandemic. BMJ Sex Reprod Health [Internet]. 2021 Feb 4;bmjsrh-2020-200976. Available from: http://jfprhc.bmj.com/content/early/2021/02/04/bmjsrh-2020-200976.abstract

22. Reynolds-Wright JJ, Johnstone A, McCabe K, Evans E, Cameron S. Adherence to treatment and prevalence of side effects when medical abortion is delivered via telemedicine: a prospective observational cohort study during COVID-19. BMJ Sex Reprod Health [Internet]. 2022 Jul 1;48(3):185. Available from: http://jfprhc.bmj.com/content/48/3/185.abstract

23. UK Government. At home early medical abortions made permanent in England and Wales [Internet]. Department of Health and Social Care; 2022 Aug. Available from: https://www.gov.uk/government/news/at-home-early-medical-abortions-made-permanent-in-england-and-wales

24. Dixon A, Reynolds-Wright JJ, Cameron ST. Uptake of long-acting reversible contraception after telemedicine delivered abortion during Covid-19. The European Journal of Contraception & Reproductive Health Care [Internet]. 2022 Jul 4;27(4):284–8. Available from: https://doi.org/10.1080/13625187.2022.2085680

25. Vianello M, Reynolds-Wright JJ, Cameron S. Self-reported contraceptive use and satisfaction among women accessing telemedicine medical abortion at the onset of the COVID-19 pandemic at 3–6-month follow-up. BMJ Sex Reprod Health [Internet]. 2023 Jan 1;49(1):21. Available from: http://jfprhc.bmj.com/content/49/1/21.abstract

For peer review only

Supplemental Figure 1: Contents of medication pack

Abortion medications	Misoprostol 800micrograms sublingual/vaginal/buccal (per patient preference)
Analgesia	Dihydrocodeine 30mg<br>Women advised to purchase their own supply of paracetamol and ibuprofen.
Confirmation of abortion	Low Sensitivity Urine Pregnancy Test 1000iu at 14 days
Contraception	Pills, patches, rings and condoms could be supplied in pack.<br>Implants were provided at clinic visit and intrauterine methods provided at rapid access clinic, with bridging method offered.
Information	Detailed step-by-step information provided as written leaflet included in pack.

For peer review only

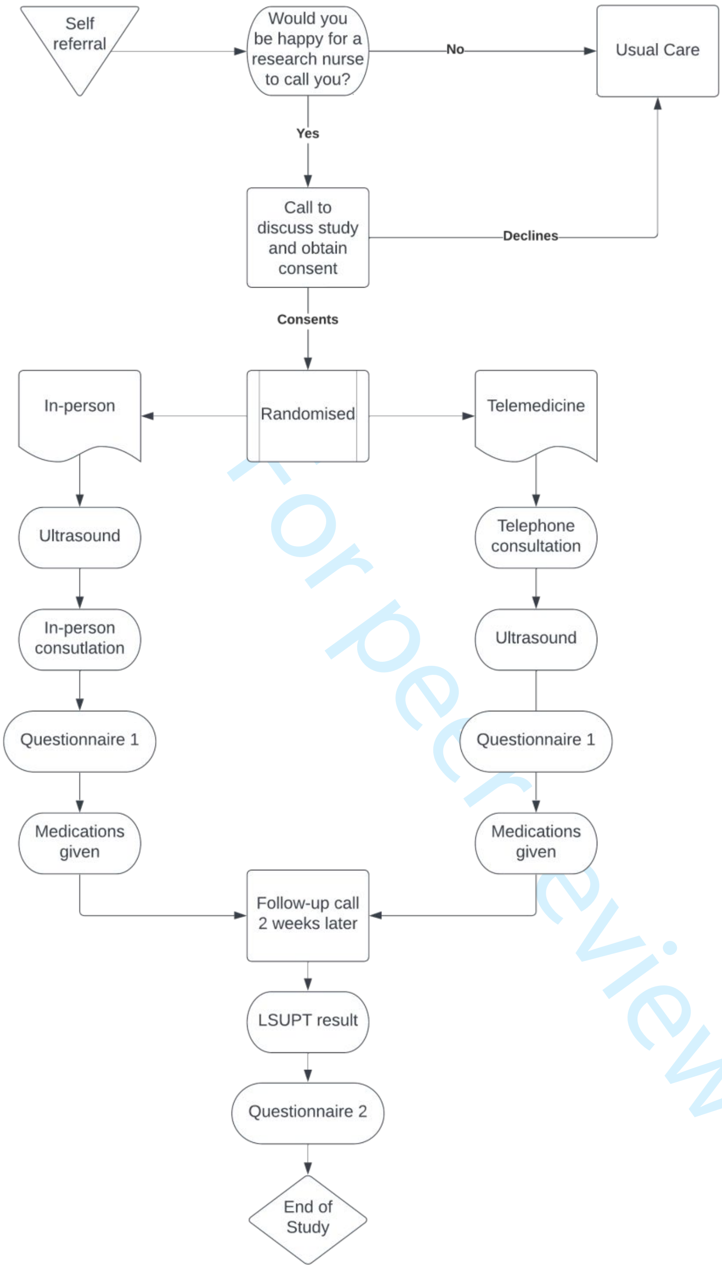

Supplemental Figure 2: Study flow chart

For peer review only

Table 1 The CONSORT supplement checklist for early MARE (MARE-C)

Section/Topic	Item No	Checklist item	Supplement for MARE	Reported on page #
**TITLE AND ABSTRACT**				
	1a	Identification as a randomized trial in the title		1
	1b	Structured summary of trial design, methods, results, and conclusions		3
**INTRODUCTION**				
Background and objectives	2a	Scientific background and explanation of rationale		5
	2b	Specific objectives or hypotheses		5
**METHODS**				
	3a	Description of trial design including allocation ratio		5,6

Trial design	3b	Important changes to methods after trial commencement (such as eligibility criteria), with reasons		n/a
Participants	4a	Eligibility criteria for participants	4a-1 Detail the range of gestational age for participants determined a priori to be included in the research, including a lower limit when applicable.	6
			4a-2 Explain the methods used to determine gestational age (e.g., physical examination, last menstrual period, ultrasonography). If ultrasonography is used, detail the type (vaginal and/or abdominal) and consider describing the criteria used for determination of gestational age.	6
	4b	Settings and locations where the data were collected		6
Interventions	5	The interventions for each group with sufficient details to allow replication, including how and when they were actually administered	Detail the medications used, including dose(s) and route(s) of administration. If more than one medication is used, state the planned time interval between medications, preferably in hours.	5,6

For peer review only - http://bmjopen.bmj.com/site/about/guidelines.xhtml

Outcomes	6a	Completely defined pre-specified primary and secondary outcome measures, including how and when they were assessed	6a-1 Outcome: Define successful medical abortion (should most commonly be considered as successful expulsion of the intrauterine pregnancy without need for surgical intervention).	7
			6a-2 Outcome: Define the types of medical abortion failure (e.g., ongoing pregnancy, incomplete abortion, participant symptoms). Continuing pregnancy should be defined as a viable pregnancy following treatment (to be differentiated from a non-viable [i.e., retained gestational sac]).	7
			6a-3 Assessment: Explain the follow-up assessments used to determine outcome (e.g., urine pregnancy test, serum pregnancy test, ultrasonography, physical exam, symptoms checklists).	8
			6a-4 Assessment: Explain the length of time planned to follow participants for determination of outcomes.	8
		Any changes to trial outcomes after		

	6b	the trial commenced, with reasons		9
Sample size	7a	How sample size was determined		8
	7b	When applicable, explanation of any interim analyses and stopping guidelines		9
**RANDOMIZATION**				
Sequence generation	8a	Method used to generate the random allocation sequence		6
	8b	Type of randomization; details of any restriction (such as blocking and block size)		6
Allocation concealment	9	Mechanism used to implement the random allocation sequence (such as sequentially numbered containers), describing any steps taken to conceal the sequence until interventions were		6

		assigned		
Implementation	10	Who generated the random allocation sequence, who enrolled participants, and who assigned participants to interventions		6
Blinding	11a	If done, who was blinded after assignment to interventions (for example, participants, care providers, those assessing outcomes) and how		n/a
	11b	If relevant, description of the similarity of interventions		n/a
Statistical methods	12a	Statistical methods used to compare groups for primary and secondary outcomes		8
	12b	Methods for additional analyses, such as subgroup analyses and adjusted analyses		n/a

RESULTS

Participant flow (a diagram is strongly recommended)	13a	For each group, the numbers of participants who were randomly assigned, received intended treatment, and were analyzed for the primary outcome	13a-1 Report the number of participants who started medical abortion treatment and the number who did not complete any follow-up for each group overall and by gestational age.*	9
			13a-2 Report the number of participants used in the denominator for outcome evaluations for each group overall and by gestational age*, which most commonly will be the number of women with any follow-up.	9,10,11
			13a-3 Include a description of the number of women who used the drug(s) as planned in the protocol (treatment adherence).	9
			13a-4 When more than one drug is used (e.g. mifepristone and a prostaglandin analog), the actual time interval between the agents should be reported, preferably in hours.	5
	13b	For each group, losses and exclusions after randomization, together with		9

For peer review only - http://bmjopen.bmj.com/site/about/guidelines.xhtml

		reasons		
Recruitment	14a	Dates defining the periods of recruitment and follow-up		9
	14b	Why the trial ended or was stopped		9
Baseline data	15	A table showing baseline demographic and clinical characteristics for each group		9
Numbers analyzed	16	For each group, number of participants (denominator) included in each analysis and whether the analysis was by original assigned groups		9,10,11
Outcomes and estimation	17a	For each primary and secondary outcome, results for each group, and the estimated effect size and its precision (such as 95% confidence interval)	17a-1 Present treatment success for each group overall and by gestational age.*	10
			17a-2 Present continuing pregnancies for each group overall and by gestational age.*	10
			17a-3 Present reasons for surgical intervention other	

			than continuing pregnancy for each group overall and by gestational age.*	10
	17b	For binary outcomes, presentation of both absolute and relative effect sizes is recommended		10
Ancillary analyses	18	Results of any other analyses performed, including subgroup analyses and adjusted analyses, distinguishing pre-specified from exploratory		10, 11
Harms	19	All important harms or unintended effects in each group		10, 11
**DISCUSSION**				
Limitations	20	Trial limitations, addressing sources of potential bias, imprecision, and, if relevant, multiplicity of analyses		12, 13
Generalizability	21	Generalizability (external validity, applicability) of the		12, 13

For peer review only - http://bmjopen.bmj.com/site/about/guidelines.xhtml

		trial findings		
Interpretation	22	Interpretation consistent with results, balancing benefits and harms, and considering other relative evidence		12, 13
**OTHER INFORMATION**				
Registration	23	Registration number and name of trial registry		9
Protocol	24	Where the full trial protocol can be accessed, if available		5
Funding	25	Sources of funding and other support (such as supply of drugs), role of funders		2

*** Present the number as a total and with detail by gestational age in days in reference to completed weeks (e.g., ≤49 days, 50–56 days, 57–63 days, 64–70 days). Note that gestations less than 49 days can be further differentiated at the discretion of the investigators.**

For peer review only - http://bmjopen.bmj.com/site/about/guidelines.xhtml

BMJ Open

Using Telemedicine to improve early medical Abortion at Home (UTAH): a randomised controlled trial to compare telemedicine with in-person consultation for early medical abortion

Journal:	*BMJ Open*
Manuscript ID	bmjopen-2023-073630.R1
Article Type:	Original research
Date Submitted by the Author:	06-Jul-2023
Complete List of Authors:	Reynolds-Wright, John; University of Edinburgh; NHS Lothian<br>Norrie, John; Edinburgh Clinical Trials Unit, University of Edinburgh No. 9, Bioquarter, Usher Institute<br>Cameron, Sharon; University of Edinburgh, Obstetrics and Gynaecology; NHS Lothian, Sexual and Reproductive Health
**Primary Subject Heading**:	Sexual health
Secondary Subject Heading:	Health services research, Evidence based practice, Public health, Reproductive medicine
Keywords:	Telemedicine < BIOTECHNOLOGY & BIOINFORMATICS, GYNAECOLOGY, OBSTETRICS, SEXUAL MEDICINE, REPRODUCTIVE MEDICINE

SCHOLARONE™ Manuscripts

I, the Submitting Author has the right to grant and does grant on behalf of all authors of the Work (as defined in the below author licence), an exclusive licence and/or a non-exclusive licence for contributions from authors who are: i) UK Crown employees; ii) where BMJ has agreed a CC-BY licence shall apply, and/or iii) in accordance with the terms applicable for US Federal Government officers or employees acting as part of their official duties; on a worldwide, perpetual, irrevocable, royalty-free basis to BMJ Publishing Group Ltd ("BMJ") its licensees and where the relevant Journal is co-owned by BMJ to the co-owners of the Journal, to publish the Work in this journal and any other BMJ products and to exploit all rights, as set out in our licence.

The Submitting Author accepts and understands that any supply made under these terms is made by BMJ to the Submitting Author unless you are acting as an employee on behalf of your employer or a postgraduate student of an affiliated institution which is paying any applicable article publishing charge ("APC") for Open Access articles. Where the Submitting Author wishes to make the Work available on an Open Access basis (and intends to pay the relevant APC), the terms of reuse of such Open Access shall be governed by a Creative Commons licence – details of these licences and which Creative Commons licence will apply to this Work are set out in our licence referred to above.

Other than as permitted in any relevant BMJ Author's Self Archiving Policies, I confirm this Work has not been accepted for publication elsewhere, is not being considered for publication elsewhere and does not duplicate material already published. I confirm all authors consent to publication of this Work and authorise the granting of this licence.

For peer review only

Using Telemedicine to improve early medical Abortion at Home (UTAH): a randomised controlled trial to compare telemedicine with in-person consultation for early medical abortion

Authors

John J Reynolds-Wright MBChB, PhD, MFSRH [1,2]
John Norrie MSc [3]
Sharon T Cameron MD FFSRH FRCOG [1,2]

Affiliations

[1]MRC Centre for Reproductive Health, University of Edinburgh, Edinburgh, UK
[2]Chalmers Centre, NHS Lothian, Edinburgh, UK
[3]Edinburgh Clinical Trials Unit, Usher Institute, University of Edinburgh, Edinburgh, UK

Correspondence to:

Dr John J Reynolds-Wright
MRC Centre for Reproductive Health
The Queen's Medical Research Institute
47 Little France Crescent
Edinburgh BioQuarter
Edinburgh
EH16 4TJ
UK
Email: John.Reynolds-Wright@ed.ac.uk

For peer review only

Abstract

Objectives: To compare telephone consultations with in-person consultations for the provision of medical abortion (using Mifepristone 200mg and Misoprostol 800microgram). We hypothesised that telemedicine consultations would be non-inferior to in-person consultations with a non-inferiority limit of 3%.

Design: Randomised controlled trial with 1:1 allocation.

Setting: Community abortion service housed within an integrated sexual and reproductive health service in Edinburgh, UK.

Participants: The trial began on January 13, 2020, but was stopped early due to COVID-19; recruitment was suspended on March 31, 2020, and was formally closed on August 31, 2021. A total of 125 participants were randomised, approximately 10% of the total planned, with 63 assigned to telemedicine and 62 to in-person consultation.

Primary and secondary outcome measures: Primary outcome: efficacy of medical abortion, defined as complete abortion without surgical intervention**.** Secondary outcomes: satisfaction with consultation type, preparedness, unscheduled contact with care, complication rate, time spent in clinical contact and uptake of long-acting contraception.

Results: Primary outcome was available for 115 participants (lost-to-follow-up telemedicine=2, in-person=8), secondary outcomes were available for 110 participants (n=5 and n= 10 in telemedicine and in-person groups did not complete questionnaires). There were no significant differences between groups in treatment efficacy (telemedicine 57/63 [90.5%], in-person 48/62 [77.4%]). However, non-inferiority was not demonstrated (+3.3% in favour of telemedicine, confidence interval -6.6% to +13.3%, lower than non-inferiority margin). There were no significant differences in most secondary outcomes, however, there was more unscheduled contact with care in the telemedicine group (12 [19%] vs 3 [5%], p=0.01). The overall time spent in clinical contact was statistically significantly lower in the telemedicine group (mean 94 [SD 24] vs 111 [24] min, p=0.0005).

Conclusions: Telemedicine for medical abortion appeared to be effective, safe, and acceptable to women, with less time spent in the clinic. However, due to the small sample size resulting from early cessation, the study was underpowered to confirm this conclusion. These findings warrant further investigation in larger scale studies.

Trial registration: ClinicalTrials.gov, NCT04139382.

For peer review only

Strengths and limitations of this study

- To our knowledge, this is the first randomised controlled trial in a high-income setting to compare telemedicine with in-person consultation for the delivery of early medical abortion care.
- Most other studies of telemedicine in abortion care have been observational cohort studies and so this trial makes a useful addition to the literature.
- Due to COVID-19, the UTAH study had to close shortly after recruitment began as telemedicine became the standard of care; as such, only 10% of the target sample was recruited and the study is underpowered for outcome assessment.

For peer review only

Introduction

In-person consultations prior to abortion can be lengthy. Women can struggle with arranging childcare or time off work to attend appointments. There is evidence from observational studies that telemedicine consultations for those seeking early medical abortion (EMA) at under 10 weeks gestation may be a safe and acceptable alternative (1–5). Additionally, the World Health Organization recommend that women can reliably self-manage much of EMA with support from a healthcare worker (6).

Prior to COVID-19 in Scotland, women who chose an EMA typically made a single visit to a clinic for a consultation, assessment of gestation, receipt of mifepristone (to be administered in clinic, as per UK legal requirements at the time) and a medication pack to take home. This contained misoprostol (to self-administer at home contraception where desired, and instructions on how to self-assess the success of the abortion using a self-performed low-sensitivity urinary pregnancy test (LSUPT, detection limit 1000 iu human chorionic gonadotrophin) at home two weeks later (7–9).

This clinic visit could last up to two hours; much of which may be time spent in the waiting room between seeing clinicians and having an ultrasound to confirm gestation. However, a significant proportion of consultation time is standardised history taking and information giving, and could be conducted via telemedicine by telephone, video call, or over the internet, rather than in-person.

Telemedicine consultations could add flexibility for women (e.g. consultation from home), and benefit services with reduced footfall in clinics and allow for more flexible staff working (including remote working). It is also possible that women may be better prepared for EMA: having a telemedicine consultation in advance of receipt of medications may mean they have had more time to process the information provided about EMA, as well as any information about post-abortion contraception options. Doing this prior to attending clinic for ultrasound (which was nearly universal prior to COVID-19) and treatment provision, may give further opportunity to clarify areas of uncertainty for abortion treatment or select a contraceptive method.

We therefore aimed to compare telemedicine with in-person consultations regarding effectiveness, safety, acceptability, and duration of consultation.

Methods

Study design

The full study protocol has been published (10). In brief, we designed a randomised controlled trial to compare telemedicine consultations, specifically a telephone call, with in-person consultations for women seeking EMA.

For peer review only

Participants

Participants were pregnant individuals seeking medical abortion at home, aged 16 or over, able to communicate in English and fulfil the requirements of the 1967 Abortion Act. In addition to being eligible to participate in the trial, women needed to be clinically eligible to receive EMA, which for the study was defined as having a viable intrauterine pregnancy determined by transabdominal or transvaginal ultrasound at or less than 9 weeks and 6 days' gestation.

Setting

The study was conducted at a single centre in the UK (Chalmers Centre, Edinburgh) that provides abortion care to a predominately urban population with some women living in more remote and rural areas. Standard care was for women to self-refer to the service by telephone and receive a subsequent in-person clinic appointment (11). At this appointment, they had a routine pre-abortion ultrasound scan to determine location and gestation of the pregnancy, followed by a consultation with a doctor or nurse to obtain medical history, discuss the chosen method of abortion and provide written informed consent. For medical abortion at home, the first medication (mifepristone 200mg) was provided orally in the clinic as per UK legal requirements at that time. Women were then provided with a medication pack for home use that included misoprostol 800 micrograms (sublingual or vaginal as per patient preference), contraceptive supplies if desired, and LSUPT and written information were provided to take home, that included instructions for using medications, wehat to expect in terms of pain and bleeding and when to seek medical attention (9,12) (see Supplemental Figure 1).

Randomisation

In the study, at the point of self-referral for abortion, the clinic administrative team asked women if they would be happy to be contacted by a research nurse. Those agreeing received a telephone call the same or next day, during which the clinical research nurses explained the study, obtained consent and randomised women using REDCap software, hosted by University of Edinburgh (13,14). The randomisation list was produced by an independent statistician. Due to the nature of the interventions, blinding was not possible.

Study groups

The 'in-person' group then went on to receive standard care as described above. Following this in-person consultation they completed a short interviewer-administered questionnaire with the research nurse in clinic.

The 'telemedicine' group received their telephone consultation using the same proforma as for in-person consultations. This was conducted by the research nurses, who has experience working in the abortion clinic and providing counselling in in-person consultations. This was done either immediately after randomisation or at

For peer review only

another time, as preferred by the woman. Following this consultation, the woman then attended the clinic for ultrasound scan, medication provision (mifepristone was administered at the appointment under clinician supervision as per legislation at the time) and completed the study questionnaire (as for in-person care group). Women in both groups received a follow up telephone call at two weeks after EMA from a research nurse to determine the LSUPT result and to administer the follow up questionnaire. These questionnaire data were combined with routinely collected clinical data on demographics, gestation as determined by pre abortion ultrasound reproductive history and deprivation score (Scottish Index of Multiple Deprivation, SIMD (15)). See Supplemental Figure 2 for overview of study flow.

Outcomes

The primary outcome of this study was to determine if EMA conducted following a telemedicine consultation is as effective (complete abortion rate without surgical intervention) as following in-person consultation. Effectiveness was chosen in accordance with the Medical Abortion Reporting of Efficacy (MARE) core outcome recommendations (16). This was determined in the first instance using self-reported LSUPT result 2 weeks after EMA, any woman with a positive LSUPT result at this point was invited to attend for follow up ultrasound to confirm/exclude continuing pregnancy. Any woman with a positive LSUPT but whose post abortion ultrasound showed an empty uterus was classed as having a complete abortion. The clinical database was reviewed at 6 weeks post-EMA to check for any admission or surgical intervention to a hospital within the region.

The secondary outcomes were total time spent in clinical contact to receive EMA (calculated as time spent in the clinic plus for the telemedicine group this also included the duration of the telephone consultation), preparedness for EMA as assessed by pre-abortion questionnaire (published as supplemental file with the open access study protocol (10)), level of satisfaction with consultation as assessed by follow up questionnaire at 2 weeks (published as supplemental file with the open access study protocol (10)), rates of unscheduled contact with abortion service and any complications resulting from abortion treatment (by case note review at 6 weeks post EMA), proportion of women who were found to be ineligible for EMA when attending clinic for pre-abortion ultrasound.

Statistical methods

The recruitment target of 1222 was calculated for the primary outcome, success of EMA, using a binary outcome non-inferiority design assuming an EMA success rate of 97% in both telemedicine and in-person groups, with 90% power, one-sided 5% level of significance, 3% non-inferiority limit, 1:1 allocation and around 10% compensation for loss to follow-up(17). Primarily due to the COVID-19 pandemic, the study only recruited 125 participants, and the observed EMA rate in the standard of care group was lower at 90% rather than the assumed 97%. Indicatively, a study with 125 evaluable participants and assumed EMA 90% could only detect a non-inferiority margin of almost 16% at 90% power and 1-sided 5% level of significance.

For peer review only - http://bmjopen.bmj.com/site/about/guidelines.xhtml

Descriptive statistics were used to characterise participants and assess comparability of the two groups at baseline.

The primary outcome of complete abortion was compared between the randomised groups (telemedicine vs in-person) by calculating the risk difference and its exact 95% confidence interval (risk difference calculator, neoweb.org.uk).

Secondary outcomes were analysed using appropriate tests depending on distribution of the data: for continuous data, we have used two sample t-tests and Mann Whitney tests; for categorical data, we have used chi-squared and Fisher Exact tests. We have not imputed any missing data for the secondary outcomes. Results will be considered statistically significant if p value <0.05. There has been no adjustment for multiple comparisons.

Ethical approval and registration

Ethical approval was granted by South East Scotland NHS Research Ethics Committee on October 28, 2019, reference: 19/SS/0111. The trial was prospectively registered at ClinicalTrials.gov on October 25, 2019 (NCT04139382).

Early cessation

The study opened to recruitment on January 13, 2020, and was suspended on March 31, 2020, in response to COVID-19 pandemic, as telemedicine became standard care. The Scottish Government subsequently conducted a public consultation to determine whether there was public support for making the COVID-19 legislative changes permanent (18). The public consultation concluded that the legislative changes allowing telemedicine should continue. This meant that there would not be a return to the previous in-person model of care that the UTAH study was designed around and so the study was formally closed on August 31, 2021. The decision to suspend and then formally close the study was made by the study team following consultation with research and development and service leads.

Patient and public involvement

We consulted Abortion Rights Edinburgh, a local abortion and women's rights activism group. They kindly provided feedback on the trial rationale, study design and study protocol prior to submission for ethical approval. They have agreed to disseminate the trial findings to their membership and via their networks.

Results

During the study period 575 people had abortions at the study centre. Of these, 128 met inclusion criteria and were approached; of these, 125 women were recruited, with 63 (50.4%) randomised to the telemedicine group and 62 (49.6%) randomised

For peer review only

to the in-person group. Treatment outcome data were available 115 women (92%). 15 women (12%) did not complete follow up questionnaires (Telemedicine = 5, In-person = 10). Those who responded to follow up contacts confirmed that they had used medications as directed with a mifepristone-misoprostol interval of 24-48 hours; however, the specific interval per participant was not collected.

Demographics are summarised in Table 1, there were no substantial differences between groups except for SIMD– the in-person group had a higher proportion of women from more deprived backgrounds.

Table 1. Demographics

	Telemedicine	In-person
BMI kg/ m2 /mean	26.4 (n = 56)	26.6 (n = 52)
Parity – total mean	1.2 (n =63)	1.6 (n=62)
Parity – live births (mean)	0.6 (n = 63)	0.98 (n = 62)
Age (mean)	28.2 (n= 63)	27.5 (n = 62)
Smoker (n/tot, %)	14/63, 22.2%	8/62, 12.9%
Gestational age weeks + days (Median)	6+6	6+5
Gestational age weeks + days (Range)	Less than 5 - 9+6	Less than 5 - 9+2
Previous live birth (%)	23 (36.5%)	29 (46.8%)
Previous abortion (%)	19 (30.2%)	15 (24.2%)
SIMD		
Unknown	1	1
1 (most deprived)	5	18
2	11	13
3	11	11
4	16	5
5 (least deprived)	19	14

Primary outcome

The complete abortion rate was high and not statistically significantly different between the two groups with a greater degree of loss to follow up in the in-person group (Table 2). The risk difference of failed abortion (continuing pregnancy) was 3.2% (CI -7.6 – 1.2). Since only around 10% of the target sample of 1220 was recruited, the study was generally substantially under powered for this outcome.

Table 2. Treatment outcome

For peer review only - http://bmjopen.bmj.com/site/about/guidelines.xhtml

	Telemedicine	In-person
**Complete abortion (negative LSUPT or empty uterus on post-treatment scan)**	Of all enrolled: 57/63 (90.5%) Excluding loss to follow up: 57/61 (93.4%)	Of all enrolled: 48/62 (77.4%) Excluding loss to follow up: 48/54 (88.9%)
**Continuing Pregnancy after treatment**	0	2/62 (3.2%)
**Miscarriage prior to treatment**	1 (1.6%)	3 (4.8%)
**Not pregnant at clinic attendance**	2 (3.2%)	1 (1.6%)
**Ectopic on ultrasound**	1 (1.6%)	0
**Lost to Follow Up**	2/63 (3.2%)	8/62 (12.9%)

Secondary outcomes

There were no complications resulting from EMA in either the in-person or telemedicine group. No patients in either group required hospitalisation, blood transfusion, emergency department visits and there were no deaths. No patients were found to be ineligible for EMA in the telemedicine group when they attended for ultrasound (i.e. gestation was not higher than expected). A higher proportion of the telemedicine group reported a positive LSUPT and had a planned further attendance for repeat ultrasound to confirm complete abortion (telemedicine 12/63, 19% vs in-person 3/62, 4.8%), however this outcome was underpowered.

Following the consultation, similar high proportions of women rated themselves as very prepared and very satisfied with the mode of consultation they received for their abortion care. See Table 3.

Table 3. Preparedness for treatment and Satisfaction with treatment

	Telemedicine N(%)	In-person
Preparedness		
Very Prepared	51 (81.0%)	44 (71.0%)
Somewhat Prepared	6 (9.5%)	8 (12.9%)
Neutral	1 (1.2%)	0
Somewhat Unprepared	0	0
Very Unprepared	0	0
Missing	5 (7.9%)	10 (15.9%)
Satisfaction		
Very Satisfied	56 (88.9%)	51 (82.3%)
Somewhat Satisfied	2 (3.2%)	1 (1.6%)
Neutral	0	0

For peer review only

Somewhat Unsatisfied	0	0
Very Unsatisfied	0	0
Missing	5 (7.9%)	10 (15.9%)

There was no statistically significant difference in the types of post-abortion contraception selected between groups when comparing long-acting reversible methods with all other methods; nor with short acting methods, condoms and no method; nor more effective methods (hormonal and intrauterine) with less effective methods. See Table 4.

Table 4. Post-abortion contraception

Table 4	Telemedicine	In-person
Copper IUD	2 (3.2%)	1 (1.6%)
Hormonal IUD	3 (4.8%)	2 (3.2%)
Subdermal Implant	2 (3.2%)	6 (9.7%)
Injectable	6 (9.5%)	0
Combined pill	11 (17.5%)	11 (17.7%)
Combined patch	0	1 (1.6%)
Progestogen only Pill	9 (14.3%)	19 (30.6%)
Condoms	14 (22.2%)	8 (12.9%)
No method	16 (25.4%)	14 (22.6%)

The mean total time spent in clinical contact with the service (telemedicine consultation and clinic visit combined for the telemedicine group; clinic visit time for the in-person group) was significantly lower ($p<0.001$) in the telemedicine group compared to the in-person group. See Table 5.

Table 5. Time spent in clinical contact

Total Minutes Contact (minutes)	*Telemedicine (teleconsult duration only) n=55*	*Telemedicine (in clinic duration only) n = 55*	Telemedicine (teleconsult AND in clinic combined) n=55	In-person (time in clinic) N=50
Mean	*24*	*69*	**94**	111
St. Dev.	*6*	*23*	**24**	24
Median	*25*	*68*	**95**	108
Range	*10-40*	*25-125*	**55-151***	60-165

*Range of phone and in clinic combined is different from ranges in first two columns as those with shortest/longest call did not have shortest/longest clinic time.

Discussion

Due to COVID-19, the UTAH study had to close shortly after recruitment began as telemedicine became the standard of care and randomising women to in-person appointments was deemed unsafe for COVID-19 transmission. As a result the study outcomes are underpowered. However, the findings that effectiveness of treatment, satisfaction and preparedness are all similar between groups reflect data from observational studies about the safety, effectiveness and acceptability of telemedicine abortion care.

The only adequately powered outcome was that time spent in pre-abortion clinical contact was significantly lower in the telemedicine group than in the in-person group. In a service utilising the model of telephone consultation followed by an ultrasound scan for gestational age and medication provision, the total clinical contact time may be even lower in practice still as in this study there were sometimes delays if telephone group patients got ‘stuck in the queue’ behind standard care patients in the clinic waiting for their ultrasound scan or medications. Models of care where an ultrasound is not required at all may reduce overall time in clinical care even further, as the ultrasound component lasted a mean of 69 minutes in this study.

The rate of contact following abortion with a positive LSUPT was higher in the telemedicine group compared to the in-person group, however this outcome was underpowered and so may not have been borne out if the study had completed recruitment. If there was a true difference, this may possibly have resulted from greater familiarity and confidence in contacting the clinic via telephone as this was the main method these participants had used prior to abortion. While all patients were advised to make contact with the clinic if they had a positive or invalid result, the telemedicine group may have been more confident in doing so, having received their prior care in this way.

The complete abortion rate in the in-person group (77%) is not statistically different from that of the telemedicine group but appears lower than reported elsewhere in the literature (19). This is likely to be due to the small numbers included in this study, meaning that this outcome is underpowered. Additionally, the participants that did not respond to follow-up contacts in this study arm did not have continuing pregnancies reported on their electronic patient record and so it is likely that they too had a complete abortion. The electronic patient record covers all the hospitals in the region and due to the geography of the area, patients uncommonly travel to neighbouring health boards.

The SIMD composition of the two study groups were different, with a greater proportion of the in-person group living in more deprived areas. This is likely to be due to chance and the small, under-recruited sample for the study.

In addition to this study, there is now a single RCT (20) in South Africa conducted since the start of COVID-19. In that study, those in the telemedicine arm had abdominal palpation from a trained nurse to assess fundal height and screen for pregnancies greater than 12 weeks’ gestation. Last Menstrual Period was used to include patients with pregnancies less than 9 weeks’ gestation. That study

For peer review only

demonstrated non-inferiority of telemedicine to standard care with regard to complete abortion rates and a preference among women receiving abortion for telemedicine. Their findings reflect the body of observational literature (2,4,5,21) regarding telemedicine abortion care, including other observational cohort studies in the UK conducted during COVID-19 (22–24). While the findings of the UTAH study were underpowered to confirm or refute non-inferiority, they also reflect these findings of Endler et al.

The legislation allowing medical abortion at home across Great Britain is now permanent (18,25), however many services in Great Britain and worldwide continue to provide abortion through in-person appointments. Moving assessment consultations to be done by telemedicine may result in time savings even if inviting the majority of patients for subsequent ultrasound.

We theorised that that this model of care may give women more time to consider contraceptive options and make a decision to select a more effective method than when confronted with this choice in a face-to-face clinic. However, in this trial as well as in subsequent observational cohort studies we have conducted at the same centre during COVID-19 (26,27), this has not appeared to be the case. Further study is needed to better link women to effective post-abortion contraception.

This is the first RCT in a high income setting to compare telemedicine with in-person appointments for the delivery of abortion care. The study was designed in line with the CONSORT and MARE guidance (16). The majority of all other studies of telemedicine in abortion care have been observational cohort studies (1–3,5) and so this trial makes a useful addition to the literature. However, as outlined above, the early cessation and under-powering of most study outcomes is the major limitation of the study.

Conclusion

Telemedicine for medical abortion appeared to be effective, safe, and acceptable to women, with less time spent in the clinic. However, due to the small sample size resulting from the early cessation of recruitment, the study was underpowered to confirm this conclusion. These findings warrant further investigation in larger scale studies.

For peer review only

Contributors

STC conceived the study. JRW, JN and STC designed the study protocol. JRW designed the study tools and led data collection. JRW, JN and STC reviewed and analysed the data. JRW wrote the initial manuscript. JRW, JN and STC all contributed to the final manuscript.

Funding

This work was supported by the Edinburgh Family Planning Trust grant number EFPT/2019/UTAH. The project was conducted from the MRC Centre for Reproductive Health, supported by the Medical Research Council (grant MR/N022556/1).

Competing interests

JRW has received an educational grant from Gedeon Richter. STC and JN have no competing interests to declare.

Data availability statement

Due to the sensitive nature of the data and small sample size, individual patient level data are not available publicly.

For peer review only

References

1. Hyland P, Raymond EG, Chong E. A direct-to-patient telemedicine abortion service in Australia: Retrospective analysis of the first 18 months. Australian and New Zealand Journal of Obstetrics and Gynaecology [Internet]. 2018 Jun 1 [cited 2020 Nov 19];58(3):335–40. Available from: https://doi.org/10.1111/ajo.12800

2. Grossman D, Grindlay K. Safety of Medical Abortion Provided Through Telemedicine Compared With In Person. Obstetrics & Gynecology [Internet]. 2017;130(4). Available from: https://journals.lww.com/greenjournal/Fulltext/2017/10000/Safety_of_Medical_Abortion_Provided_Through.16.aspx

3. Grossman D, Grindlay K, Buchacker T, Lane K, Blanchard K. Effectiveness and Acceptability of Medical Abortion Provided Through Telemedicine. Obstetrics & Gynecology [Internet]. 2011;118(2 Part 1). Available from: https://journals.lww.com/greenjournal/Fulltext/2011/08000/Effectiveness_and_Acceptability_of_Medical.14.aspx

4. Wiebe ER. Use of telemedicine for providing medical abortion. Int J Gynaecol Obstet. 2014 Feb;124(2):177–8.

5. Kohn JE, Snow JL, Grossman D, Thompson TA, Seymour JW, Simons HR. Introduction of telemedicine for medication abortion: Changes in service delivery patterns in two U.S. states. Contraception [Internet]. 2021 Mar 1;103(3):151–6. Available from: https://www.sciencedirect.com/science/article/pii/S0010782420304583

6. World Health Organization. Expanding health worker roles for safe abortion in the first trimester of pregnancy [Internet]. Geneva; 2016 May. Report No.: WHO/RHR/16.02. Available from: https://www.who.int/reproductivehealth/publications/unsafe_abortion/health-worker-roles-safe-abortion-1st-trimester-summary/en/

7. Chief Medical Officer for Scotland. Abortion - Improvement to existing services - Approval for misoprostol to be taken at home [Internet]. SGHD/CMO(2017)14 Oct 26, 2017. Available from: https://www.sehd.scot.nhs.uk/cmo/CMO(2017)14.pdf

8. Cameron ST, Glasier A, Johnstone A, Dewart H, Campbell A. Can women determine the success of early medical termination of pregnancy themselves? Contraception [Internet]. 2015 Jan 1;91(1):6–11. Available from: http://www.sciencedirect.com/science/article/pii/S0010782414006908

9. Millar SL, Cameron ST. Comparison of two low-sensitivity urine pregnancy tests for confirming the success of early medical abortion. BMJ Sex Reprod Health [Internet]. 2018 Jan 1;44(1):54. Available from: http://jfprhc.bmj.com/content/44/1/54.abstract

For peer review only

10. Reynolds-Wright JJ, Norrie J, Cameron ST. UTAH: Using Telemedicine to improve early medical Abortion at Home: a protocol for a randomised controlled trial comparing face-to-face with telephone consultations for women seeking early medical abortion. BMJ Open [Internet]. 2021 Jun 1;11(6):e046628. Available from: http://bmjopen.bmj.com/content/11/6/e046628.abstract

11. Low ST, Chen ZE, Cameron S. Women's experiences of self-referral to an abortion service: qualitative study. BMJ Sex Reprod Health [Internet]. 2020 Apr 7;bmjsrh-2019-200568. Available from: http://jfprhc.bmj.com/content/early/2020/04/08/bmjsrh-2019-200568.abstract

12. Finch RE, McGeechan K, Johnstone A, Cameron S. Impact of self-administration of misoprostol for early medical abortion: a prospective observational cohort study. BMJ Sex Reprod Health [Internet]. 2019 Oct 1;45(4):296. Available from: http://jfprhc.bmj.com/content/45/4/296.abstract

13. Harris PA, Taylor R, Minor BL, Elliott V, Fernandez M, O'Neal L, et al. The REDCap consortium: Building an international community of software platform partners. Journal of Biomedical Informatics [Internet]. 2019 Jul 1;95:103208. Available from: https://www.sciencedirect.com/science/article/pii/S1532046419301261

14. Harris PA, Taylor R, Thielke R, Payne J, Gonzalez N, Conde JG. Research electronic data capture (REDCap)—A metadata-driven methodology and workflow process for providing translational research informatics support. Journal of Biomedical Informatics [Internet]. 2009 Apr 1;42(2):377–81. Available from: https://www.sciencedirect.com/science/article/pii/S1532046408001226

15. Scottish Government. Scottish Index of Multiple Deprivation (SIMD) [Internet]. 2020. Available from: www.simd.scot

16. Creinin MD, Chen MJ. Medical abortion reporting of efficacy: the MARE guidelines. Contraception. 2016;

17. Sealed Enveloped Ltd. Power calculator for binary outcome non-inferiority trial [Internet]. 2012. Available from: https://www.sealedenvelope.com/power/binary-noninferior/

18. Scottish Government. Early medical abortion at home - future arrangements: consutlation analysis [Internet]. 2021 Jun. Available from: https://www.gov.scot/publications/future-arrangements-early-medical-abortion-home-consultation-analysis/pages/1/

19. Kahn JG, Becker BJ, MacIsaa L, Amory JK, Neuhaus J, Olkin I, et al. The efficacy of medical abortion: a meta-analysis. Contraception. 2000 Jan;61(1):29–40.

20. Endler M, Petro G, Gemzell Danielsson K, Grossman D, Gomperts R, Weinryb M, et al. A telemedicine model for abortion in South Africa: a randomised, controlled, non-inferiority trial. The Lancet [Internet]. 2022 Aug

For peer review only

27;400(10353):670–9. Available from: https://www.sciencedirect.com/science/article/pii/S014067362201474X

21. Upadhyay UD, Koenig LR, Meckstroth KR. Safety and Efficacy of Telehealth Medication Abortions in the US During the COVID-19 Pandemic. JAMA Network Open [Internet]. 2021 Aug 24 [cited 2021 Oct 14];4(8):e2122320–e2122320. Available from: https://doi.org/10.1001/jamanetworkopen.2021.22320

22. Aiken A, Lohr P, Lord J, Ghosh N, Starling J. Effectiveness, safety and acceptability of no-test medical abortion (termination of pregnancy) provided via telemedicine: a national cohort study. BJOG: An International Journal of Obstetrics & Gynaecology [Internet]. 2021 Feb 18 [cited 2021 Jun 25];n/a(n/a). Available from: https://doi.org/10.1111/1471-0528.16668

23. Reynolds-Wright JJ, Johnstone A, McCabe K, Evans E, Cameron S. Telemedicine medical abortion at home under 12 weeks' gestation: a prospective observational cohort study during the COVID-19 pandemic. BMJ Sex Reprod Health [Internet]. 2021 Feb 4;bmjsrh-2020-200976. Available from: http://jfprhc.bmj.com/content/early/2021/02/04/bmjsrh-2020-200976.abstract

24. Reynolds-Wright JJ, Johnstone A, McCabe K, Evans E, Cameron S. Adherence to treatment and prevalence of side effects when medical abortion is delivered via telemedicine: a prospective observational cohort study during COVID-19. BMJ Sex Reprod Health [Internet]. 2022 Jul 1;48(3):185. Available from: http://jfprhc.bmj.com/content/48/3/185.abstract

25. UK Government. At home early medical abortions made permanent in England and Wales [Internet]. Department of Health and Social Care; 2022 Aug. Available from: https://www.gov.uk/government/news/at-home-early-medical-abortions-made-permanent-in-england-and-wales

26. Dixon A, Reynolds-Wright JJ, Cameron ST. Uptake of long-acting reversible contraception after telemedicine delivered abortion during Covid-19. The European Journal of Contraception & Reproductive Health Care [Internet]. 2022 Jul 4;27(4):284–8. Available from: https://doi.org/10.1080/13625187.2022.2085680

27. Vianello M, Reynolds-Wright JJ, Cameron S. Self-reported contraceptive use and satisfaction among women accessing telemedicine medical abortion at the onset of the COVID-19 pandemic at 3–6-month follow-up. BMJ Sex Reprod Health [Internet]. 2023 Jan 1;49(1):21. Available from: http://jfprhc.bmj.com/content/49/1/21.abstract

For peer review only

Supplemental Figure 1: Contents of medication pack

Abortion medications	Misoprostol 800micrograms sublingual/vaginal/buccal (per patient preference)
Analgesia	Dihydrocodeine 30mg<br>Women advised to purchase their own supply of paracetamol and ibuprofen.
Confirmation of abortion	Low Sensitivity Urine Pregnancy Test 1000iu at 14 days
Contraception	Pills, patches, rings and condoms could be supplied in pack.<br>Implants were provided at clinic visit and intrauterine methods provided at rapid access clinic, with bridging method offered.
Information	Detailed step-by-step information provided as written leaflet included in pack.

For peer review only

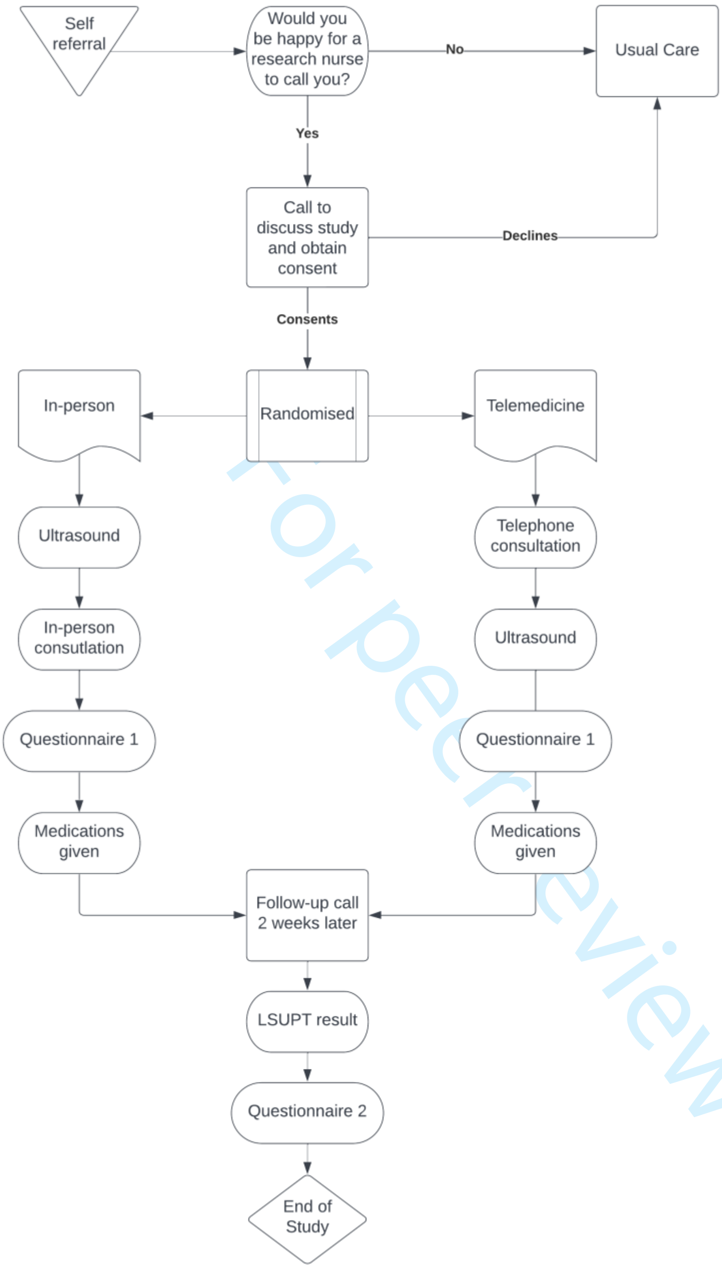

Supplemental Figure 2: Study flow chart

Table 1 The CONSORT supplement checklist for early MARE (MARE-C)

Section/Topic	Item No	Checklist item	Supplement for MARE	Reported on page #
**TITLE AND ABSTRACT**				
	1a	Identification as a randomized trial in the title		1
	1b	Structured summary of trial design, methods, results, and conclusions		3
**INTRODUCTION**				
Background and objectives	2a	Scientific background and explanation of rationale		5
	2b	Specific objectives or hypotheses		5
**METHODS**				
	3a	Description of trial design including allocation ratio		5,6

Trial design	3b	Important changes to methods after trial commencement (such as eligibility criteria), with reasons		n/a
Participants	4a	Eligibility criteria for participants	4a-1 Detail the range of gestational age for participants determined a priori to be included in the research, including a lower limit when applicable.	6
			4a-2 Explain the methods used to determine gestational age (e.g., physical examination, last menstrual period, ultrasonography). If ultrasonography is used, detail the type (vaginal and/or abdominal) and consider describing the criteria used for determination of gestational age.	6
	4b	Settings and locations where the data were collected		6
Interventions	5	The interventions for each group with sufficient details to allow replication, including how and when they were actually administered	Detail the medications used, including dose(s) and route(s) of administration. If more than one medication is used, state the planned time interval between medications, preferably in hours.	5,6

For peer review only - http://bmjopen.bmj.com/site/about/guidelines.xhtml

Outcomes	6a	Completely defined pre-specified primary and secondary outcome measures, including how and when they were assessed	6a-1 Outcome: Define successful medical abortion (should most commonly be considered as successful expulsion of the intrauterine pregnancy without need for surgical intervention).	7
			6a-2 Outcome: Define the types of medical abortion failure (e.g., ongoing pregnancy, incomplete abortion, participant symptoms). Continuing pregnancy should be defined as a viable pregnancy following treatment (to be differentiated from a non-viable [i.e., retained gestational sac]).	7
			6a-3 Assessment: Explain the follow-up assessments used to determine outcome (e.g., urine pregnancy test, serum pregnancy test, ultrasonography, physical exam, symptoms checklists).	8
			6a-4 Assessment: Explain the length of time planned to follow participants for determination of outcomes.	8
		Any changes to trial outcomes after		

For peer review only - http://bmjopen.bmj.com/site/about/guidelines.xhtml

	6b	the trial commenced, with reasons		9
Sample size	7a	How sample size was determined		8
	7b	When applicable, explanation of any interim analyses and stopping guidelines		9
**RANDOMIZATION**				
Sequence generation	8a	Method used to generate the random allocation sequence		6
	8b	Type of randomization; details of any restriction (such as blocking and block size)		6
Allocation concealment	9	Mechanism used to implement the random allocation sequence (such as sequentially numbered containers), describing any steps taken to conceal the sequence until interventions were		6

		assigned		
Implementation	10	Who generated the random allocation sequence, who enrolled participants, and who assigned participants to interventions		6
Blinding	11a	If done, who was blinded after assignment to interventions (for example, participants, care providers, those assessing outcomes) and how		n/a
	11b	If relevant, description of the similarity of interventions		n/a
Statistical methods	12a	Statistical methods used to compare groups for primary and secondary outcomes		8
	12b	Methods for additional analyses, such as subgroup analyses and adjusted analyses		n/a

**RESULTS**				
Participant flow (a diagram is strongly recommended)	13a	For each group, the numbers of participants who were randomly assigned, received intended treatment, and were analyzed for the primary outcome	13a-1 Report the number of participants who started medical abortion treatment and the number who did not complete any follow-up for each group overall and by gestational age.*	9
			13a-2 Report the number of participants used in the denominator for outcome evaluations for each group overall and by gestational age*, which most commonly will be the number of women with any follow-up.	9,10,11
			13a-3 Include a description of the number of women who used the drug(s) as planned in the protocol (treatment adherence).	9
			13a-4 When more than one drug is used (e.g. mifepristone and a prostaglandin analog), the actual time interval between the agents should be reported, preferably in hours.	5
	13b	For each group, losses and exclusions after randomization, together with		9

For peer review only - http://bmjopen.bmj.com/site/about/guidelines.xhtml

		reasons		
Recruitment	14a	Dates defining the periods of recruitment and follow-up		9
	14b	Why the trial ended or was stopped		9
Baseline data	15	A table showing baseline demographic and clinical characteristics for each group		9
Numbers analyzed	16	For each group, number of participants (denominator) included in each analysis and whether the analysis was by original assigned groups		9,10,11
Outcomes and estimation	17a	For each primary and secondary outcome, results for each group, and the estimated effect size and its precision (such as 95% confidence interval)	17a-1 Present treatment success for each group overall and by gestational age.*	10
			17a-2 Present continuing pregnancies for each group overall and by gestational age.*	10
			17a-3 Present reasons for surgical intervention other	

			than continuing pregnancy for each group overall and by gestational age.*	10
	17b	For binary outcomes, presentation of both absolute and relative effect sizes is recommended		10
Ancillary analyses	18	Results of any other analyses performed, including subgroup analyses and adjusted analyses, distinguishing pre-specified from exploratory		10, 11
Harms	19	All important harms or unintended effects in each group		10, 11
**DISCUSSION**				
Limitations	20	Trial limitations, addressing sources of potential bias, imprecision, and, if relevant, multiplicity of analyses		12, 13
Generalizability	21	Generalizability (external validity, applicability) of the		12, 13

		trial findings		
Interpretation	22	Interpretation consistent with results, balancing benefits and harms, and considering other relative evidence		12, 13
**OTHER INFORMATION**				
Registration	23	Registration number and name of trial registry		9
Protocol	24	Where the full trial protocol can be accessed, if available		5
Funding	25	Sources of funding and other support (such as supply of drugs), role of funders		2

*** Present the number as a total and with detail by gestational age in days in reference to completed weeks (e.g., ≤49 days, 50–56 days, 57–63 days, 64–70 days). Note that gestations less than 49 days can be further differentiated at the discretion of the investigators.**

For peer review only - http://bmjopen.bmj.com/site/about/guidelines.xhtml

CONSERVE-CONSORT Extension: 04/07/2023 – UTAH Study					
**Item**	**Item Title**	**Description**			**Page No.**
I.	Extenuating Circumstances	Describe the circumstances and how they constitute extenuating circumstances.			8
II.	Important Modifications	a. Describe how the modifications are important modifications.			8 (cessation rather than modification)
		b. Describe the impacts and mitigating strategies, including their rationale and implications for the trial.			(see below)
		c. Provide a modification timeline.			n/a - cessation
III.	Responsible Parties	State who planned, reviewed and approved the modifications.			8
IV.	Interim data	If modifications were informed by trial data, describe how the interim data were used, including whether they were examined by study group, and whether the individuals reviewing the data were blinded to the treatment allocation.			n/a
**CONSORT Number and Item**		For each row, if important modifications occurred check "direct impact" and/or "mitigating strategy" and describe the changes in the trial manuscript or supplement. Check "no change" for items that are unaffected in the extenuating circumstance.			**Page No.**
		**No Change**	**Impact***	**Mitigating Strategy****	
1	Title and abstract	X			
2	Introduction	X			
3	Methods: Trial Design	X			
4	Methods: Participants	X			
5	Methods: Interventions	X			
6	Methods: Outcomes	X			
7	Methods: Sample Size	X			
8-10	Methods: Randomisation	X			

For peer review only

11	Methods: Blinding	X			
12	Methods: Statistical methods	X			
13	Results: Participant flow	X			
14	Results: Recruitment		X		8
15	Results: Baseline data	X			
16	Results: Numbers analysed		X		8,9
	Results: Outcomes and estimation		X		9,11,12
18	Results: Ancillary analyses		X		9,11,12
19	Results: Harms	X			
20	Discussion: Limitations		X		9,11,12
21	Discussion: Generalisability	X			
	Other information: Registration	X			
24	Other information: Protocol	X			
25	Other information: Funding	X			

*Aspects of the trial that are directly affected or changed by the extenuating circumstance and are not under the control of investigators, sponsor or funder.
**Aspects of the trial that are modified by the study investigators, sponsor or funder to respond to the extenuating circumstance or manage the direct impacts on the trial.

For peer review only

BMJ Open

Using Telemedicine to improve early medical Abortion at Home (UTAH): a randomised controlled trial to compare telemedicine with in-person consultation for early medical abortion

Journal:	*BMJ Open*
Manuscript ID	bmjopen-2023-073630.R2
Article Type:	Original research
Date Submitted by the Author:	28-Aug-2023
Complete List of Authors:	Reynolds-Wright, John; University of Edinburgh; NHS Lothian<br>Norrie, John; Edinburgh Clinical Trials Unit, University of Edinburgh No. 9, Bioquarter, Usher Institute<br>Cameron, Sharon; University of Edinburgh, Obstetrics and Gynaecology; NHS Lothian, Sexual and Reproductive Health
**Primary Subject Heading**:	Sexual health
Secondary Subject Heading:	Health services research, Evidence based practice, Public health, Reproductive medicine, Obstetrics and gynaecology
Keywords:	Telemedicine < BIOTECHNOLOGY & BIOINFORMATICS, GYNAECOLOGY, OBSTETRICS, SEXUAL MEDICINE, REPRODUCTIVE MEDICINE

SCHOLARONE™
Manuscripts

I, the Submitting Author has the right to grant and does grant on behalf of all authors of the Work (as defined in the below author licence), an exclusive licence and/or a non-exclusive licence for contributions from authors who are: i) UK Crown employees; ii) where BMJ has agreed a CC-BY licence shall apply, and/or iii) in accordance with the terms applicable for US Federal Government officers or employees acting as part of their official duties; on a worldwide, perpetual, irrevocable, royalty-free basis to BMJ Publishing Group Ltd ("BMJ") its licensees and where the relevant Journal is co-owned by BMJ to the co-owners of the Journal, to publish the Work in this journal and any other BMJ products and to exploit all rights, as set out in our licence.

The Submitting Author accepts and understands that any supply made under these terms is made by BMJ to the Submitting Author unless you are acting as an employee on behalf of your employer or a postgraduate student of an affiliated institution which is paying any applicable article publishing charge ("APC") for Open Access articles. Where the Submitting Author wishes to make the Work available on an Open Access basis (and intends to pay the relevant APC), the terms of reuse of such Open Access shall be governed by a Creative Commons licence – details of these licences and which Creative Commons licence will apply to this Work are set out in our licence referred to above.

Other than as permitted in any relevant BMJ Author's Self Archiving Policies, I confirm this Work has not been accepted for publication elsewhere, is not being considered for publication elsewhere and does not duplicate material already published. I confirm all authors consent to publication of this Work and authorise the granting of this licence.

For peer review only

Using Telemedicine to improve early medical Abortion at Home (UTAH): a randomised controlled trial to compare telemedicine with in-person consultation for early medical abortion

Authors

John J Reynolds-Wright MBChB, PhD, MFSRH [1,2]
John Norrie MSc [3]
Sharon T Cameron MD FFSRH FRCOG [1,2]

Affiliations

[1]MRC Centre for Reproductive Health, University of Edinburgh, Edinburgh, UK
[2]Chalmers Centre, NHS Lothian, Edinburgh, UK
[3]Edinburgh Clinical Trials Unit, Usher Institute, University of Edinburgh, Edinburgh, UK

Correspondence to:

Dr John J Reynolds-Wright
MRC Centre for Reproductive Health
The Queen's Medical Research Institute
47 Little France Crescent
Edinburgh BioQuarter
Edinburgh
EH16 4TJ
UK
Email: John.Reynolds-Wright@ed.ac.uk

For peer review only

Abstract

Objectives: To compare telephone consultations with in-person consultations for the provision of medical abortion (using Mifepristone 200mg and Misoprostol 800microgram). We hypothesised that telemedicine consultations would be non-inferior to in-person consultations with a non-inferiority limit of 3%.

Design: Randomised controlled trial with 1:1 allocation.

Setting: Community abortion service housed within an integrated sexual and reproductive health service in Edinburgh, UK.

Participants: The trial began on January 13, 2020, but was stopped early due to COVID-19; recruitment was suspended on March 31, 2020, and was formally closed on August 31, 2021. A total of 125 participants were randomised, approximately 10% of the total planned, with 63 assigned to telemedicine and 62 to in-person consultation.

Primary and secondary outcome measures: Primary outcome: efficacy of medical abortion, defined as complete abortion without surgical intervention**.** Secondary outcomes: satisfaction with consultation type, preparedness, unscheduled contact with care, complication rate, time spent in clinical contact and uptake of long-acting contraception.

Results: Primary outcome was available for 115 participants (lost-to-follow-up telemedicine=2, in-person=8), secondary outcomes were available for 110 participants (n=5 and n= 10 in telemedicine and in-person groups did not complete questionnaires). There were no significant differences between groups in treatment efficacy (telemedicine 57/63 [90.5%], in-person 48/62 [77.4%]). However, non-inferiority was not demonstrated (+3.3% in favour of telemedicine, confidence interval -6.6% to +13.3%, lower than non-inferiority margin). There were no significant differences in most secondary outcomes, however, there was more unscheduled contact with care in the telemedicine group (12 [19%] vs 3 [5%], p=0.01). The overall time spent in clinical contact was statistically significantly lower in the telemedicine group (mean 94 [SD 24] vs 111 [24] min, p=0.0005).

Conclusions: Telemedicine for medical abortion appeared to be effective, safe, and acceptable to women, with less time spent in the clinic. However, due to the small sample size resulting from early cessation, the study was underpowered to confirm this conclusion. These findings warrant further investigation in larger scale studies.

Trial registration: ClinicalTrials.gov, NCT04139382.

For peer review only

Strengths and limitations of this study

- To our knowledge, this is the first randomised controlled trial in a high-income setting to compare telemedicine with in-person consultation for the delivery of early medical abortion care.
- Most other studies of telemedicine in abortion care have been observational cohort studies and so this trial makes a useful addition to the literature.
- Due to COVID-19, the UTAH study had to close shortly after recruitment began as telemedicine became the standard of care; as such, only 10% of the target sample was recruited and the study is underpowered for outcome assessment.

For peer review only

Introduction

In-person consultations prior to abortion can be lengthy. Women can struggle with arranging childcare or time off work to attend appointments. There is evidence from observational studies that telemedicine consultations for those seeking early medical abortion (EMA) at under 10 weeks gestation may be a safe and acceptable alternative (1–5). Additionally, the World Health Organization recommend that women can reliably self-manage much of EMA with support from a healthcare worker (6).

Prior to COVID-19 in Scotland, women who chose an EMA typically made a single visit to a clinic for a consultation, assessment of gestation, receipt of mifepristone (to be administered in clinic, as per UK legal requirements at the time) and a medication pack to take home. This contained misoprostol (to self-administer at home contraception where desired, and instructions on how to self-assess the success of the abortion using a self-performed low-sensitivity urinary pregnancy test (LSUPT, detection limit 1000 iu human chorionic gonadotrophin) at home two weeks later (7–9).

This clinic visit could last up to two hours; much of which may be time spent in the waiting room between seeing clinicians and having an ultrasound to confirm gestation. However, a significant proportion of consultation time is standardised history taking and information giving, and could be conducted via telemedicine by telephone, video call, or over the internet, rather than in-person.

Telemedicine consultations could add flexibility for women (e.g. consultation from home), and benefit services with reduced footfall in clinics and allow for more flexible staff working (including remote working). It is also possible that women may be better prepared for EMA: having a telemedicine consultation in advance of receipt of medications may mean they have had more time to process the information provided about EMA, as well as any information about post-abortion contraception options. Doing this prior to attending clinic for ultrasound (which was nearly universal prior to COVID-19) and treatment provision, may give further opportunity to clarify areas of uncertainty for abortion treatment or select a contraceptive method.

We therefore aimed to compare telemedicine with in-person consultations regarding effectiveness, safety, acceptability, and duration of consultation.

Methods

Study design

The full study protocol has been published (10). In brief, we designed a randomised controlled trial to compare telemedicine consultations, specifically a telephone call, with in-person consultations for women seeking EMA.

For peer review only - http://bmjopen.bmj.com/site/about/guidelines.xhtml

Participants

Participants were pregnant individuals seeking medical abortion at home, aged 16 or over, able to communicate in English and fulfil the requirements of the 1967 Abortion Act. In addition to being eligible to participate in the trial, women needed to be clinically eligible to receive EMA, which for the study was defined as having a viable intrauterine pregnancy determined by transabdominal or transvaginal ultrasound at or less than 9 weeks and 6 days' gestation.

Setting

The study was conducted at a single centre in the UK (Chalmers Centre, Edinburgh) that provides abortion care to a predominately urban population with some women living in more remote and rural areas. Standard care was for women to self-refer to the service by telephone and receive a subsequent in-person clinic appointment (11). At this appointment, they had a routine pre-abortion ultrasound scan to determine location and gestation of the pregnancy, followed by a consultation with a doctor or nurse to obtain medical history, discuss the chosen method of abortion and provide written informed consent. Counselling about post-abortion contraception was offered routinely to all patients. For medical abortion at home, the first medication (mifepristone 200mg) was provided orally in the clinic as per UK legal requirements at that time. Women were then provided with a medication pack for home use that included misoprostol 800 micrograms (sublingual or vaginal as per patient preference), contraceptive supplies if desired, and LSUPT and written information were provided to take home, that included instructions for using medications, what to expect in terms of pain and bleeding and when to seek medical attention, including calling the clinic for further advice (9,12) (see Supplemental Figure 1).

Randomisation

In the study, at the point of self-referral for abortion, the clinic administrative team asked women if they would be happy to be contacted by a research nurse. Those agreeing received a telephone call the same or next day, during which the clinical research nurses explained the study, obtained consent and randomised women using REDCap software, hosted by University of Edinburgh (13,14). The randomisation list was produced by an independent statistician. Due to the nature of the interventions, blinding was not possible.

Study groups

The 'in-person' group then went on to receive standard care as described above. Following this in-person consultation they completed a short interviewer-administered questionnaire with the research nurse in clinic.

The 'telemedicine' group received their telephone consultation using the same proforma as for in-person consultations, including discussion of post-abortion contraception. This was conducted by the research nurses, who has experience

For peer review only

working in the abortion clinic and providing counselling in in-person consultations. This was done either immediately after randomisation or at another time, as preferred by the woman. Following this consultation, the woman then attended the clinic for ultrasound scan, medication provision (mifepristone was administered at the appointment under clinician supervision as per legislation at the time) and completed the study questionnaire (as for in-person care group). Women in both groups received a follow up telephone call at two weeks after EMA from a research nurse to determine the LSUPT result and to administer the follow up questionnaire. These questionnaire data were combined with routinely collected clinical data on demographics, gestation as determined by pre abortion ultrasound reproductive history and deprivation score (Scottish Index of Multiple Deprivation, SIMD (15)). See Supplemental Figure 2 for overview of study flow.

Outcomes

The primary outcome of this study was to determine if EMA conducted following a telemedicine consultation is as effective (complete abortion rate without surgical intervention) as following in-person consultation. Effectiveness was chosen in accordance with the Medical Abortion Reporting of Efficacy (MARE) core outcome recommendations (16). This was determined in the first instance using self-reported LSUPT result 2 weeks after EMA, any woman with a positive LSUPT result at this point was invited to attend for follow up ultrasound to confirm/exclude continuing pregnancy. Any woman with a positive LSUPT but whose post abortion ultrasound showed an empty uterus was classed as having a complete abortion. The clinical database was reviewed at 6 weeks post-EMA to check for any admission or surgical intervention to a hospital within the region.

The secondary outcomes were total time spent in clinical contact to receive EMA (calculated as time spent in the clinic plus for the telemedicine group this also included the duration of the telephone consultation), preparedness for EMA as assessed by pre-abortion questionnaire (published as supplemental file with the open access study protocol (10)), level of satisfaction with consultation as assessed by follow up questionnaire at 2 weeks (published as supplemental file with the open access study protocol (10)), rates of unscheduled contact with abortion service (initiated by the patient or emergency attendance) and any complications resulting from abortion treatment (by case note review at 6 weeks post EMA), proportion of women who were found to be ineligible for EMA when attending clinic for pre-abortion ultrasound.

Statistical methods

The recruitment target of 1222 was calculated for the primary outcome, success of EMA, using a binary outcome non-inferiority design assuming an EMA success rate of 97% in both telemedicine and in-person groups, with 90% power, one-sided 5% level of significance, 3% non-inferiority limit, 1:1 allocation and around 10% compensation for loss to follow-up(17). Primarily due to the COVID-19 pandemic, the study only recruited 125 participants, and the observed EMA rate in the standard of care group was lower at 90% rather than the assumed 97%. Indicatively, a study

with 125 evaluable participants and assumed EMA 90% could only detect a non-inferiority margin of almost 16% at 90% power and 1-sided 5% level of significance.

Descriptive statistics were used to characterise participants and assess comparability of the two groups at baseline.

The primary outcome of complete abortion was compared between the randomised groups (telemedicine vs in-person) by calculating the risk difference and its exact 95% confidence interval (risk difference calculator, neoweb.org.uk).

Secondary outcomes were analysed using appropriate tests depending on distribution of the data: for continuous data, we have used two sample t-tests and Mann Whitney tests; for categorical data, we have used chi-squared and Fisher Exact tests. We have not imputed any missing data for the secondary outcomes. Results will be considered statistically significant if p value <0.05. There has been no adjustment for multiple comparisons.

Ethical approval and registration

Ethical approval was granted by South East Scotland NHS Research Ethics Committee on October 28, 2019, reference: 19/SS/0111. The trial was prospectively registered at ClinicalTrials.gov on October 25, 2019 (NCT04139382).

Early cessation

The study opened to recruitment on January 13, 2020, and was suspended on March 31, 2020, in response to COVID-19 pandemic, as telemedicine became standard care. The Scottish Government subsequently conducted a public consultation to determine whether there was public support for making the COVID-19 legislative changes permanent (18). The public consultation concluded that the legislative changes allowing telemedicine should continue. This meant that there would not be a return to the previous in-person model of care that the UTAH study was designed around and so the study was formally closed on August 31, 2021. The decision to suspend and then formally close the study was made by the study team following consultation with research and development and service leads.

Patient and public involvement

We consulted Abortion Rights Edinburgh, a local abortion and women's rights activism group. They kindly provided feedback on the trial rationale, study design and study protocol prior to submission for ethical approval. They have agreed to disseminate the trial findings to their membership and via their networks.

Results

For peer review only

During the study period 575 people had abortions at the study centre. Of these, 128 met inclusion criteria and were approached; of these, 125 women were recruited, with 63 (50.4%) randomised to the telemedicine group and 62 (49.6%) randomised to the in-person group. Treatment outcome data were available 115 women (92%). 15 women (12%) did not complete follow up questionnaires (Telemedicine = 5, In-person = 10). Those who responded to follow up contacts confirmed that they had used medications as directed with a mifepristone-misoprostol interval of 24-48 hours; however, the specific interval per participant was not collected.

Demographics are summarised in Table 1, there were no substantial differences between groups except for SIMD– the in-person group had a higher proportion of women from more deprived backgrounds.

Table 1. Demographics

	Telemedicine	In-person
BMI kg/ m2 /mean	26.4 (n = 56)	26.6 (n = 52)
Parity – total mean	1.2 (n =63)	1.6 (n=62)
Parity – live births (mean)	0.6 (n = 63)	0.98 (n = 62)
Age (mean)	28.2 (n= 63)	27.5 (n = 62)
Smoker (n/tot, %)	14/63, 22.2%	8/62, 12.9%
**Gestational age weeks + days (Median)**	6+6	6+5
**Gestational age weeks + days (Range)**	Less than 5 - 9+6	Less than 5 - 9+2
**Previous live birth (%)**	23 (36.5%)	29 (46.8%)
**Previous abortion (%)**	19 (30.2%)	15 (24.2%)
SIMD		
Unknown	1	1
1 (most deprived)	5	18
2	11	13
3	11	11
4	16	5
5 (least deprived)	19	14

Primary outcome

The complete abortion rate was high and not statistically significantly different between the two groups with a greater degree of loss to follow up in the in-person group (Table 2). The risk difference of failed abortion (continuing pregnancy) was

For peer review only

3.2% (CI -7.6 – 1.2). Since only around 10% of the target sample of 1220 was recruited, the study was generally substantially under powered for this outcome.

Table 2. Treatment outcome

	Telemedicine	In-person
**Complete abortion (negative LSUPT or empty uterus on post-treatment scan)**	Of all enrolled: 57/63 (90.5%)<br>Excluding loss to follow up: 57/61 (93.4%)	Of all enrolled: 48/62 (77.4%)<br>Excluding loss to follow up: 48/54 (88.9%)
**Continuing Pregnancy after treatment**	0	2/62 (3.2%)
**Miscarriage prior to treatment**	1 (1.6%)	3 (4.8%)
**Not pregnant at clinic attendance**	2 (3.2%)	1 (1.6%)
**Ectopic on ultrasound**	1 (1.6%)	0
**Lost to Follow Up**	2/63 (3.2%)	8/62 (12.9%)

Secondary outcomes

There were no complications resulting from EMA in either the in-person or telemedicine group. No patients in either group required hospitalisation, blood transfusion, emergency department visits and there were no deaths. No patients were found to be ineligible for EMA in the telemedicine group when they attended for ultrasound (i.e. gestation was not higher than expected). A higher proportion of the telemedicine group reported a positive LSUPT and had a planned further attendance for repeat ultrasound to confirm complete abortion (telemedicine 12/63, 19% vs in-person 3/62, 4.8%), however this outcome was underpowered.

Following the consultation, similar high proportions of women rated themselves as very prepared and very satisfied with the mode of consultation they received for their abortion care. See Table 3.

Table 3. Preparedness for treatment and satisfaction with treatment

	Telemedicine N(%)	In-person
Preparedness		
Very Prepared	51 (81.0%)	44 (71.0%)
Somewhat Prepared	6 (9.5%)	8 (12.9%)
Neutral	1 (1.2%)	0
Somewhat Unprepared	0	0
Very Unprepared	0	0
Missing	5 (7.9%)	10 (15.9%)
Satisfaction		
Very Satisfied	56 (88.9%)	51 (82.3%)

Somewhat Satisfied	2 (3.2%)	1 (1.6%)
Neutral	0	0
Somewhat Unsatisfied	0	0
Very Unsatisfied	0	0
Missing	5 (7.9%)	10 (15.9%)

There was no statistically significant difference in the types of post-abortion contraception selected between groups when comparing long-acting reversible methods with all other methods; nor with short acting methods, condoms and no method; nor more effective methods (hormonal and intrauterine) with less effective methods. See Table 4.

Table 4. Post-abortion contraception

Table 4	Telemedicine	In-person
Copper IUD	2 (3.2%)	1 (1.6%)
Hormonal IUD	3 (4.8%)	2 (3.2%)
Subdermal Implant	2 (3.2%)	6 (9.7%)
Injectable	6 (9.5%)	0
Combined pill	11 (17.5%)	11 (17.7%)
Combined patch	0	1 (1.6%)
Progestogen only Pill	9 (14.3%)	19 (30.6%)
Condoms	14 (22.2%)	8 (12.9%)
No method	16 (25.4%)	14 (22.6%)

The mean total time spent in clinical contact with the service (telemedicine consultation and clinic visit combined for the telemedicine group; clinic visit time for the in-person group) was significantly lower ($p<0.001$) in the telemedicine group compared to the in-person group. See Table 5.

Table 5. Time spent in clinical contact

Total Minutes Contact (minutes)	*Telemedicine (teleconsult duration only) n=55*	*Telemedicine (in clinic duration only) n = 55*	Telemedicine (teleconsult AND in clinic combined) n=55	In-person (time in clinic) N=50
Mean	*24*	*69*	**94**	111
St. Dev.	*6*	*23*	**24**	24
Median	*25*	*68*	**95**	108
Range	*10-40*	*25-125*	**55-151***	60-165

*Range of phone and in clinic combined is different from ranges in first two columns as those with shortest/longest call did not have shortest/longest clinic time.

For peer review only

Discussion

Due to COVID-19, the UTAH study had to close shortly after recruitment began as telemedicine became the standard of care and randomising women to in-person appointments was deemed unsafe for COVID-19 transmission. As a result the study outcomes are underpowered. However, the findings that effectiveness of treatment, satisfaction and preparedness are all similar between groups reflect data from observational studies about the safety, effectiveness and acceptability of telemedicine abortion care.

The only adequately powered outcome was that time spent in pre-abortion clinical contact was significantly lower in the telemedicine group than in the in-person group. In a service utilising the model of telephone consultation followed by an ultrasound scan for gestational age and medication provision, the total clinical contact time may be even lower in practice still as in this study there were sometimes delays if telephone group patients got 'stuck in the queue' behind standard care patients in the clinic waiting for their ultrasound scan or medications. Models of care where an ultrasound is not required at all may reduce overall time in clinical care even further, as the ultrasound component lasted a mean of 69 minutes in this study.

The rate of contact following abortion with a positive LSUPT was higher in the telemedicine group compared to the in-person group, however this outcome was underpowered and so may not have been borne out if the study had completed recruitment. If there was a true difference, this may possibly have resulted from greater familiarity and confidence in contacting the clinic via telephone as this was the main method these participants had used prior to abortion. While all patients were advised to make contact with the clinic if they had a positive or invalid result, the telemedicine group may have been more confident in doing so, having received their prior care in this way.

The complete abortion rate in the in-person group (77%) is not statistically different from that of the telemedicine group but appears lower than reported elsewhere in the literature (19). This is likely to be due to the small numbers included in this study, meaning that this outcome is underpowered. Additionally, the participants that did not respond to follow-up contacts in this study arm did not have continuing pregnancies reported on their electronic patient record and so it is likely that they too had a complete abortion. The electronic patient record covers all the hospitals in the region and due to the geography of the area, patients uncommonly travel to neighbouring health boards.

The SIMD composition of the two study groups were different, with a greater proportion of the in-person group living in more deprived areas. This is likely to be due to chance and the small, under-recruited sample for the study.

In addition to this study, there is now a single RCT (20) in South Africa conducted since the start of COVID-19. In that study, those in the telemedicine arm had abdominal palpation from a trained nurse to assess fundal height and screen for

For peer review only

pregnancies greater than 12 weeks' gestation. Last Menstrual Period was used to include patients with pregnancies less than 9 weeks' gestation. That study demonstrated non-inferiority of telemedicine to standard care with regard to complete abortion rates and a preference among women receiving abortion for telemedicine. Their findings reflect the body of observational literature (2,4,5,21) regarding telemedicine abortion care, including other observational cohort studies in the UK conducted during COVID-19 (22–24). While the findings of the UTAH study were underpowered to confirm or refute non-inferiority, they also reflect these findings of Endler et al.

The legislation allowing medical abortion at home across Great Britain is now permanent (18,25), however many services in Great Britain and worldwide continue to provide abortion through in-person appointments. Moving assessment consultations to be done by telemedicine may result in time savings even if inviting the majority of patients for subsequent ultrasound.

We theorised that that this model of care may give women more time to consider contraceptive options and make a decision to select a more effective method than when confronted with this choice in a face-to-face clinic. However, in this trial as well as in subsequent observational cohort studies we have conducted at the same centre during COVID-19 (26,27), this has not appeared to be the case. Further study is needed to better link women to effective post-abortion contraception.

This is the first RCT in a high income setting to compare telemedicine with in-person appointments for the delivery of abortion care. The study was designed in line with the CONSORT and MARE guidance (16). The majority of all other studies of telemedicine in abortion care have been observational cohort studies (1–3,5) and so this trial makes a useful addition to the literature. However, as outlined above, the early cessation and under-powering of most study outcomes is the major limitation of the study.

Conclusion

Telemedicine for medical abortion appeared to be effective, safe, and acceptable to women, with less time spent in the clinic. However, due to the small sample size resulting from the early cessation of recruitment, the study was underpowered to confirm this conclusion. These findings warrant further investigation in larger scale studies.

For peer review only

Contributors

STC conceived the study. JRW, JN and STC designed the study protocol. JRW designed the study tools and led data collection. JRW, JN and STC reviewed and analysed the data. JRW wrote the initial manuscript. JRW, JN and STC all contributed to the final manuscript.

Funding

This work was supported by the Edinburgh Family Planning Trust grant number EFPT/2019/UTAH. The project was conducted from the MRC Centre for Reproductive Health, supported by the Medical Research Council (grant MR/N022556/1).

Competing interests

JRW has received an educational grant from Gedeon Richter. STC and JN have no competing interests to declare.

Data availability statement

Due to the sensitive nature of the data and small sample size, individual patient level data are not available publicly.

For peer review only

References

1. Hyland P, Raymond EG, Chong E. A direct-to-patient telemedicine abortion service in Australia: Retrospective analysis of the first 18 months. Australian and New Zealand Journal of Obstetrics and Gynaecology [Internet]. 2018 Jun 1 [cited 2020 Nov 19];58(3):335–40. Available from: https://doi.org/10.1111/ajo.12800

2. Grossman D, Grindlay K. Safety of Medical Abortion Provided Through Telemedicine Compared With In Person. Obstetrics & Gynecology [Internet]. 2017;130(4). Available from: https://journals.lww.com/greenjournal/Fulltext/2017/10000/Safety_of_Medical_Abortion_Provided_Through.16.aspx

3. Grossman D, Grindlay K, Buchacker T, Lane K, Blanchard K. Effectiveness and Acceptability of Medical Abortion Provided Through Telemedicine. Obstetrics & Gynecology [Internet]. 2011;118(2 Part 1). Available from: https://journals.lww.com/greenjournal/Fulltext/2011/08000/Effectiveness_and_Acceptability_of_Medical.14.aspx

4. Wiebe ER. Use of telemedicine for providing medical abortion. Int J Gynaecol Obstet. 2014 Feb;124(2):177–8.

5. Kohn JE, Snow JL, Grossman D, Thompson TA, Seymour JW, Simons HR. Introduction of telemedicine for medication abortion: Changes in service delivery patterns in two U.S. states. Contraception [Internet]. 2021 Mar 1;103(3):151–6. Available from: https://www.sciencedirect.com/science/article/pii/S0010782420304583

6. World Health Organization. Expanding health worker roles for safe abortion in the first trimester of pregnancy [Internet]. Geneva; 2016 May. Report No.: WHO/RHR/16.02. Available from: https://www.who.int/reproductivehealth/publications/unsafe_abortion/health-worker-roles-safe-abortion-1st-trimester-summary/en/

7. Chief Medical Officer for Scotland. Abortion - Improvement to existing services - Approval for misoprostol to be taken at home [Internet]. SGHD/CMO(2017)14 Oct 26, 2017. Available from: https://www.sehd.scot.nhs.uk/cmo/CMO(2017)14.pdf

8. Cameron ST, Glasier A, Johnstone A, Dewart H, Campbell A. Can women determine the success of early medical termination of pregnancy themselves? Contraception [Internet]. 2015 Jan 1;91(1):6–11. Available from: http://www.sciencedirect.com/science/article/pii/S0010782414006908

9. Millar SL, Cameron ST. Comparison of two low-sensitivity urine pregnancy tests for confirming the success of early medical abortion. BMJ Sex Reprod Health [Internet]. 2018 Jan 1;44(1):54. Available from: http://jfprhc.bmj.com/content/44/1/54.abstract

For peer review only

10. Reynolds-Wright JJ, Norrie J, Cameron ST. UTAH: Using Telemedicine to improve early medical Abortion at Home: a protocol for a randomised controlled trial comparing face-to-face with telephone consultations for women seeking early medical abortion. BMJ Open [Internet]. 2021 Jun 1;11(6):e046628. Available from: http://bmjopen.bmj.com/content/11/6/e046628.abstract

11. Low ST, Chen ZE, Cameron S. Women's experiences of self-referral to an abortion service: qualitative study. BMJ Sex Reprod Health [Internet]. 2020 Apr 7;bmjsrh-2019-200568. Available from: http://jfprhc.bmj.com/content/early/2020/04/08/bmjsrh-2019-200568.abstract

12. Finch RE, McGeechan K, Johnstone A, Cameron S. Impact of self-administration of misoprostol for early medical abortion: a prospective observational cohort study. BMJ Sex Reprod Health [Internet]. 2019 Oct 1;45(4):296. Available from: http://jfprhc.bmj.com/content/45/4/296.abstract

13. Harris PA, Taylor R, Minor BL, Elliott V, Fernandez M, O'Neal L, et al. The REDCap consortium: Building an international community of software platform partners. Journal of Biomedical Informatics [Internet]. 2019 Jul 1;95:103208. Available from: https://www.sciencedirect.com/science/article/pii/S1532046419301261

14. Harris PA, Taylor R, Thielke R, Payne J, Gonzalez N, Conde JG. Research electronic data capture (REDCap)—A metadata-driven methodology and workflow process for providing translational research informatics support. Journal of Biomedical Informatics [Internet]. 2009 Apr 1;42(2):377–81. Available from: https://www.sciencedirect.com/science/article/pii/S1532046408001226

15. Scottish Government. Scottish Index of Multiple Deprivation (SIMD) [Internet]. 2020. Available from: www.simd.scot

16. Creinin MD, Chen MJ. Medical abortion reporting of efficacy: the MARE guidelines. Contraception. 2016;

17. Sealed Enveloped Ltd. Power calculator for binary outcome non-inferiority trial [Internet]. 2012. Available from: https://www.sealedenvelope.com/power/binary-noninferior/

18. Scottish Government. Early medical abortion at home - future arrangements: consutlation analysis [Internet]. 2021 Jun. Available from: https://www.gov.scot/publications/future-arrangements-early-medical-abortion-home-consultation-analysis/pages/1/

19. Kahn JG, Becker BJ, MacIsaa L, Amory JK, Neuhaus J, Olkin I, et al. The efficacy of medical abortion: a meta-analysis. Contraception. 2000 Jan;61(1):29–40.

20. Endler M, Petro G, Gemzell Danielsson K, Grossman D, Gomperts R, Weinryb M, et al. A telemedicine model for abortion in South Africa: a randomised, controlled, non-inferiority trial. The Lancet [Internet]. 2022 Aug

For peer review only

27;400(10353):670–9. Available from: https://www.sciencedirect.com/science/article/pii/S014067362201474X

21. Upadhyay UD, Koenig LR, Meckstroth KR. Safety and Efficacy of Telehealth Medication Abortions in the US During the COVID-19 Pandemic. JAMA Network Open [Internet]. 2021 Aug 24 [cited 2021 Oct 14];4(8):e2122320–e2122320. Available from: https://doi.org/10.1001/jamanetworkopen.2021.22320

22. Aiken A, Lohr P, Lord J, Ghosh N, Starling J. Effectiveness, safety and acceptability of no-test medical abortion (termination of pregnancy) provided via telemedicine: a national cohort study. BJOG: An International Journal of Obstetrics & Gynaecology [Internet]. 2021 Feb 18 [cited 2021 Jun 25];n/a(n/a). Available from: https://doi.org/10.1111/1471-0528.16668

23. Reynolds-Wright JJ, Johnstone A, McCabe K, Evans E, Cameron S. Telemedicine medical abortion at home under 12 weeks' gestation: a prospective observational cohort study during the COVID-19 pandemic. BMJ Sex Reprod Health [Internet]. 2021 Feb 4;bmjsrh-2020-200976. Available from: http://jfprhc.bmj.com/content/early/2021/02/04/bmjsrh-2020-200976.abstract

24. Reynolds-Wright JJ, Johnstone A, McCabe K, Evans E, Cameron S. Adherence to treatment and prevalence of side effects when medical abortion is delivered via telemedicine: a prospective observational cohort study during COVID-19. BMJ Sex Reprod Health [Internet]. 2022 Jul 1;48(3):185. Available from: http://jfprhc.bmj.com/content/48/3/185.abstract

25. UK Government. At home early medical abortions made permanent in England and Wales [Internet]. Department of Health and Social Care; 2022 Aug. Available from: https://www.gov.uk/government/news/at-home-early-medical-abortions-made-permanent-in-england-and-wales

26. Dixon A, Reynolds-Wright JJ, Cameron ST. Uptake of long-acting reversible contraception after telemedicine delivered abortion during Covid-19. The European Journal of Contraception & Reproductive Health Care [Internet]. 2022 Jul 4;27(4):284–8. Available from: https://doi.org/10.1080/13625187.2022.2085680

27. Vianello M, Reynolds-Wright JJ, Cameron S. Self-reported contraceptive use and satisfaction among women accessing telemedicine medical abortion at the onset of the COVID-19 pandemic at 3–6-month follow-up. BMJ Sex Reprod Health [Internet]. 2023 Jan 1;49(1):21. Available from: http://jfprhc.bmj.com/content/49/1/21.abstract

For peer review only

Supplemental Figure 1: Contents of medication pack

Abortion medications	Misoprostol 800micrograms sublingual/vaginal/buccal (per patient preference)
Analgesia	Dihydrocodeine 30mg<br>Women advised to purchase their own supply of paracetamol and ibuprofen.
Confirmation of abortion	Low Sensitivity Urine Pregnancy Test 1000iu at 14 days
Contraception	Pills, patches, rings and condoms could be supplied in pack.<br>Implants were provided at clinic visit and intrauterine methods provided at rapid access clinic, with bridging method offered.
Information	Detailed step-by-step information provided as written leaflet included in pack.

For peer review only

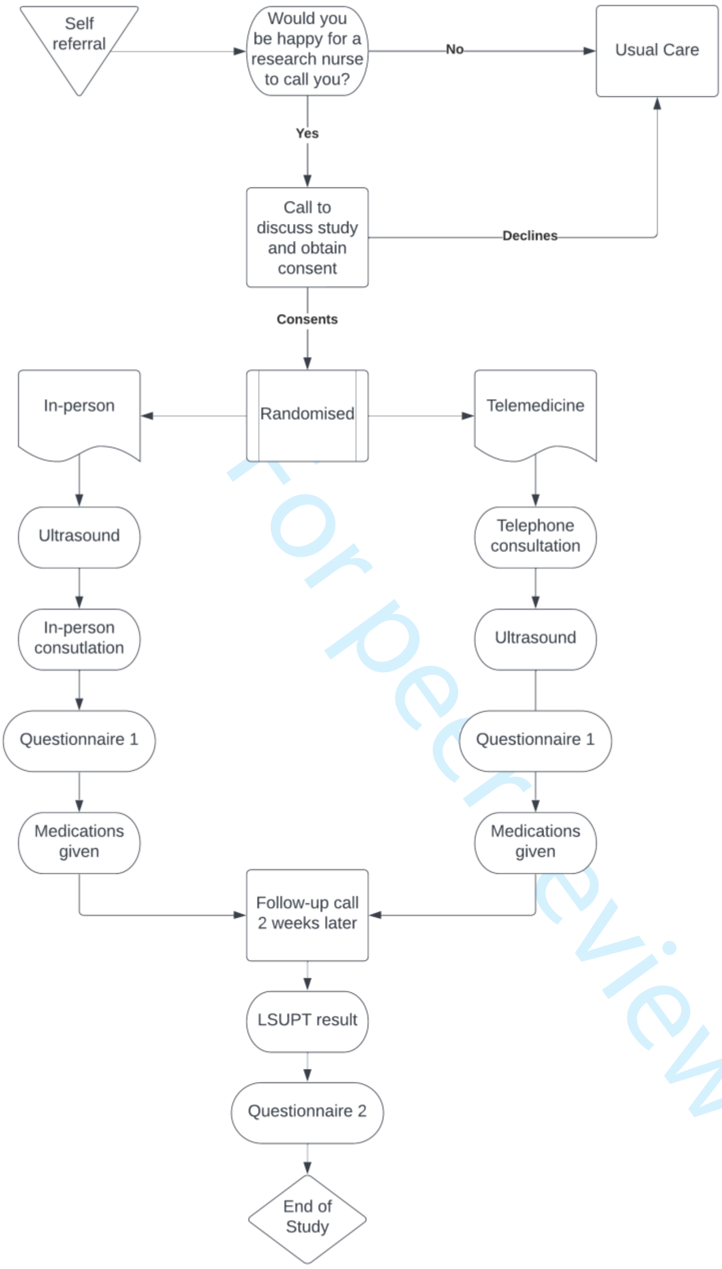

Supplemental Figure 2: Study flow chart

Table 1 The CONSORT supplement checklist for early MARE (MARE-C)

Section/Topic	Item No	Checklist item	Supplement for MARE	Reported on page #
**TITLE AND ABSTRACT**				
	1a	Identification as a randomized trial in the title		1
	1b	Structured summary of trial design, methods, results, and conclusions		3
**INTRODUCTION**				
Background and objectives	2a	Scientific background and explanation of rationale		5
	2b	Specific objectives or hypotheses		5
**METHODS**				
	3a	Description of trial design including allocation ratio		5,6

Trial design	3b	Important changes to methods after trial commencement (such as eligibility criteria), with reasons		n/a
Participants	4a	Eligibility criteria for participants	4a-1 Detail the range of gestational age for participants determined a priori to be included in the research, including a lower limit when applicable.	6
			4a-2 Explain the methods used to determine gestational age (e.g., physical examination, last menstrual period, ultrasonography). If ultrasonography is used, detail the type (vaginal and/or abdominal) and consider describing the criteria used for determination of gestational age.	6
	4b	Settings and locations where the data were collected		6
Interventions	5	The interventions for each group with sufficient details to allow replication, including how and when they were actually administered	Detail the medications used, including dose(s) and route(s) of administration. If more than one medication is used, state the planned time interval between medications, preferably in hours.	5,6

For peer review only - http://bmjopen.bmj.com/site/about/guidelines.xhtml

Outcomes	6a	Completely defined pre-specified primary and secondary outcome measures, including how and when they were assessed	6a-1 Outcome: Define successful medical abortion (should most commonly be considered as successful expulsion of the intrauterine pregnancy without need for surgical intervention).	7
			6a-2 Outcome: Define the types of medical abortion failure (e.g., ongoing pregnancy, incomplete abortion, participant symptoms). Continuing pregnancy should be defined as a viable pregnancy following treatment (to be differentiated from a non-viable [i.e., retained gestational sac]).	7
			6a-3 Assessment: Explain the follow-up assessments used to determine outcome (e.g., urine pregnancy test, serum pregnancy test, ultrasonography, physical exam, symptoms checklists).	8
			6a-4 Assessment: Explain the length of time planned to follow participants for determination of outcomes.	8
		Any changes to trial outcomes after		

For peer review only - http://bmjopen.bmj.com/site/about/guidelines.xhtml

	6b	the trial commenced, with reasons		9
Sample size	7a	How sample size was determined		8
	7b	When applicable, explanation of any interim analyses and stopping guidelines		9
**RANDOMIZATION**				
Sequence generation	8a	Method used to generate the random allocation sequence		6
	8b	Type of randomization; details of any restriction (such as blocking and block size)		6
Allocation concealment	9	Mechanism used to implement the random allocation sequence (such as sequentially numbered containers), describing any steps taken to conceal the sequence until interventions were		6

For peer review only - http://bmjopen.bmj.com/site/about/guidelines.xhtml

		assigned		
Implementation	10	Who generated the random allocation sequence, who enrolled participants, and who assigned participants to interventions		6
Blinding	11a	If done, who was blinded after assignment to interventions (for example, participants, care providers, those assessing outcomes) and how		n/a
	11b	If relevant, description of the similarity of interventions		n/a
Statistical methods	12a	Statistical methods used to compare groups for primary and secondary outcomes		8
	12b	Methods for additional analyses, such as subgroup analyses and adjusted analyses		n/a

RESULTS

Participant flow (a diagram is strongly recommended)	13a	For each group, the numbers of participants who were randomly assigned, received intended treatment, and were analyzed for the primary outcome	13a-1 Report the number of participants who started medical abortion treatment and the number who did not complete any follow-up for each group overall and by gestational age.*	9
			13a-2 Report the number of participants used in the denominator for outcome evaluations for each group overall and by gestational age*, which most commonly will be the number of women with any follow-up.	9,10,11
			13a-3 Include a description of the number of women who used the drug(s) as planned in the protocol (treatment adherence).	9
			13a-4 When more than one drug is used (e.g. mifepristone and a prostaglandin analog), the actual time interval between the agents should be reported, preferably in hours.	5
	13b	For each group, losses and exclusions after randomization, together with		9

For peer review only - http://bmjopen.bmj.com/site/about/guidelines.xhtml

		reasons		
Recruitment	14a	Dates defining the periods of recruitment and follow-up		9
	14b	Why the trial ended or was stopped		9
Baseline data	15	A table showing baseline demographic and clinical characteristics for each group		9
Numbers analyzed	16	For each group, number of participants (denominator) included in each analysis and whether the analysis was by original assigned groups		9,10,11
Outcomes and estimation	17a	For each primary and secondary outcome, results for each group, and the estimated effect size and its precision (such as 95% confidence interval)	17a-1 Present treatment success for each group overall and by gestational age.*	10
			17a-2 Present continuing pregnancies for each group overall and by gestational age.*	10
			17a-3 Present reasons for surgical intervention other	

			than continuing pregnancy for each group overall and by gestational age.*	10
	17b	For binary outcomes, presentation of both absolute and relative effect sizes is recommended		10
Ancillary analyses	18	Results of any other analyses performed, including subgroup analyses and adjusted analyses, distinguishing pre-specified from exploratory		10, 11
Harms	19	All important harms or unintended effects in each group		10, 11
**DISCUSSION**				
Limitations	20	Trial limitations, addressing sources of potential bias, imprecision, and, if relevant, multiplicity of analyses		12, 13
Generalizability	21	Generalizability (external validity, applicability) of the		12, 13

		trial findings		
Interpretation	22	Interpretation consistent with results, balancing benefits and harms, and considering other relative evidence		12, 13
**OTHER INFORMATION**				
Registration	23	Registration number and name of trial registry		9
Protocol	24	Where the full trial protocol can be accessed, if available		5
Funding	25	Sources of funding and other support (such as supply of drugs), role of funders		2

*** Present the number as a total and with detail by gestational age in days in reference to completed weeks (e.g., ≤49 days, 50–56 days, 57–63 days, 64–70 days). Note that gestations less than 49 days can be further differentiated at the discretion of the investigators.**

For peer review only - http://bmjopen.bmj.com/site/about/guidelines.xhtml

CONSERVE-CONSORT Extension: 04/07/2023 – UTAH Study

Item	Item Title	Description	Page No.
I.	Extenuating Circumstances	Describe the circumstances and how they constitute extenuating circumstances.	8
II.	Important Modifications	a. Describe how the modifications are important modifications.	8 (cessation rather than modification)
		b. Describe the impacts and mitigating strategies, including their rationale and implications for the trial.	(see below)
		c. Provide a modification timeline.	n/a - cessation
III.	Responsible Parties	State who planned, reviewed and approved the modifications.	8
IV.	Interim data	If modifications were informed by trial data, describe how the interim data were used, including whether they were examined by study group, and whether the individuals reviewing the data were blinded to the treatment allocation.	n/a

**CONSORT Number and Item**		For each row, if important modifications occurred check "direct impact" and/or "mitigating strategy" and describe the changes in the trial manuscript or supplement. Check "no change" for items that are unaffected in the extenuating circumstance.			**Page No.**
		**No Change**	**Impact***	**Mitigating Strategy****	
1	Title and abstract	X			
2	Introduction	X			
3	Methods: Trial Design	X			
4	Methods: Participants	X			
5	Methods: Interventions	X			
6	Methods: Outcomes	X			
7	Methods: Sample Size	X			
8-10	Methods: Randomisation	X			

For peer review only

11	Methods: Blinding	X			
12	Methods: Statistical methods	X			
13	Results: Participant flow	X			
14	Results: Recruitment		X		8
15	Results: Baseline data	X			
16	Results: Numbers analysed		X		8,9
	Results: Outcomes and estimation		X		9,11,12
18	Results: Ancillary analyses		X		9,11,12
19	Results: Harms	X			
20	Discussion: Limitations		X		9,11,12
21	Discussion: Generalisability	X			
	Other information: Registration	X			
24	Other information: Protocol	X			
25	Other information: Funding	X			

*Aspects of the trial that are directly affected or changed by the extenuating circumstance and are not under the control of investigators, sponsor or funder.
**Aspects of the trial that are modified by the study investigators, sponsor or funder to respond to the extenuating circumstance or manage the direct impacts on the trial.
